# SIRT3 deregulation is linked to mitochondrial dysfunction in Alzheimer's disease

**DOI:** 10.1111/acel.12679

**Published:** 2017-11-11

**Authors:** Junghee Lee, Yunha Kim, Tian Liu, Yu Jin Hwang, Seung Jae Hyeon, Hyeonjoo Im, Kyungeun Lee, Victor E. Alvarez, Ann C. McKee, Soo‐Jong Um, Manwook Hur, Inhee Mook‐Jung, Neil W. Kowall, Hoon Ryu

**Affiliations:** ^1^ VA Boston Healthcare System Boston MA 02130 USA; ^2^ Alzheimer's Disease Center and Department of Neurology Boston University School of Medicine Boston MA 02118 USA; ^3^ Laboratory for Neuronal Gene Regulation and Epigenetics Center for NeuroMedicine Brain Science Institute Korea Institute of Science and Technology Seoul 02792 South Korea; ^4^ Advanced Analysis Center Korea Institute of Science and Technology Seoul 02792 South Korea; ^5^ Bedford VA Medical Center Bedford MA 01730 USA; ^6^ Department of Integrative Bioscience and Biotechnology Sejong University Seoul 05006 South Korea; ^7^ Department of Biochemistry Yonsei University College of Medicine Seoul 03722 South Korea; ^8^ Departments of Biochemistry and Biomedical Sciences Seoul National University College of Medicine Seoul 03080 South Korea; ^9^ Present address: USF Health Byrd Alzheimer's Institute and Department of Molecular Medicine University of South Florida College of Medicine Tampa FL 33613 USA

**Keywords:** Alzheimer's disease, gene expression, mitochondria, p53, SIRT3

## Abstract

Alzheimer's disease (AD) is the leading cause of dementia in the elderly. Despite decades of study, effective treatments for AD are lacking. Mitochondrial dysfunction has been closely linked to the pathogenesis of AD, but the relationship between mitochondrial pathology and neuronal damage is poorly understood. Sirtuins (SIRT, silent mating type information regulation 2 homolog in yeast) are NAD‐dependent histone deacetylases involved in aging and longevity. The objective of this study was to investigate the relationship between SIRT3 and mitochondrial function and neuronal activity in AD. SIRT3 mRNA and protein levels were significantly decreased in AD cerebral cortex, and Ac‐p53 K320 was significantly increased in AD mitochondria. SIRT3 prevented p53‐induced mitochondrial dysfunction and neuronal damage in a deacetylase activity‐dependent manner. Notably, mitochondrially targeted p53 (mito‐p53) directly reduced mitochondria DNA‐encoded ND2 and ND4 gene expression resulting in increased reactive oxygen species (ROS) and reduced mitochondrial oxygen consumption. ND2 and ND4 gene expressions were significantly decreased in patients with AD. p53‐ChIP analysis verified the presence of p53‐binding elements in the human mitochondrial genome and increased p53 occupancy of mitochondrial DNA in AD. SIRT3 overexpression restored the expression of ND2 and ND4 and improved mitochondrial oxygen consumption by repressing mito‐p53 activity. Our results indicate that SIRT3 dysfunction leads to p53‐mediated mitochondrial and neuronal damage in AD. Therapeutic modulation of SIRT3 activity may ameliorate mitochondrial pathology and neurodegeneration in AD.

## Introduction

Alzheimer's disease (AD), the most common age‐dependent neurodegenerative disease, is characterized by irreversible memory loss and cognitive decline. Patients with AD may exhibit a range of behavioral and psychological symptoms including mood changes. Degeneration of subcortical cholinergic basal forebrain neurons may lead to the dysfunction of gamma‐amino butyric acid (GABA)ergic and glutamatergic neuronal systems, and degeneration of subcortical serotonergic and aminergic nuclei may also contribute to AD symptoms (Coyle *et al*., 1983; Canter *et al*., 2016). Accumulations of intracellular and extracellular protein aggregates, that is neurofibrillary tangles containing phosphorylated Tau protein and beta‐amyloid plaques, directly contribute to neuronal dysfunction and neurodegeneration and the development of progressive development of dementia. Many therapeutic approaches have targeted cholinergic restoration and beta‐amyloid removal, but unfortunately therapeutics directed at these targets have had very limited success to date (Wolfe, 2002; Canter *et al*., 2016).

A growing body of evidence suggests that epigenetic modifications contribute to AD pathogenesis (Lee & Ryu, 2010). Epigenetic changes encompass an array of molecular modifications to both DNA and chromatin, including transcription factors and cofactors. SIRTs (mammalian homolog of silent mating type information regulation 2 homolog in yeast) are a family of epigenetic mediators that play various functions in aging, chromatin integrity, metabolic regulation, and longevity (Kim *et al*., 2007; Hirschey *et al*., 2010). Among sirtuins, SIRT1 has been mostly widely studied and its level is changed in AD brains (Shi *et al*., 2005; Someya *et al*., 2010; Kim *et al*., 2011).

SIRT3, the most abundant sirtuin in the brain, is localized to the mitochondrial inner membrane and matrix and nucleus of neurons (Onyango *et al*., 2002). It regulates mitochondrial activity by ROS in many cell types and modulates CREB phosphorylation and fat metabolism (Shi *et al*., 2005; Hirschey *et al*., 2010; Kim *et al*., 2010). Importantly, SIRT3 has an essential role in enhancing the mitochondrial antioxidant glutathione, which could contribute to reduce aging in mammals (Someya *et al*., 2010). Recently, it is reported that SIRT3 acts as a pro‐survival factor in neurons exposed to NMDA‐induced excitotoxic injury (Kim *et al*., 2011). SIRT3 expression is elevated in animals subjected to calorie restriction, suggesting that SIRT3 may play a role in lifespan prolongation. There are no differences in the frequency of SIRT3 gene methylation in young vs. old human subjects or in patients with AD (Silva *et al*., 2008).

p53, a tumor suppressor protein and a transcription factor, translocates to the inner mitochondrial matrix under normal condition and in response to DNA damage (Marchenko *et al*., 2000; Mahyar‐Roemer *et al*., 2004). Mitochondrial p53 forms an inhibitory complex with Bcl2 and Bcl‐xL resulting in the release of cytochrome c from mitochondria to the cytosol and activation of caspases (Mihara *et al*., 2003). The translocation of p53 to mitochondria alters mitochondrial membrane potential (Zhao *et al*., 2005). Interestingly, amyloid beta 42 activates p53 transcriptional activity and upregulates p53 expression in AD, suggesting involvement of the p53 pathway in AD (Ohyagi *et al*., 2005). Notably, p53 deficiency significantly decreases oxidative stress and activates neuroprotective pathways (Barone *et al*., 2012; Fiorini *et al*., 2012). Although many reports show an association between p53 and AD, little is known about how p53 participates in neuronal mitochondrial activity and mitochondria‐encoded gene regulation in AD.

In spite of the abundance of SIRT3 and p53 in the neuronal mitochondria, their function in the brain remains to be investigated. The mitochondria‐associated senescence domain of p53 can interact with SIRT3 resulting in growth arrest (Li *et al*., 2010). Because p53 is elevated in AD brain and SIRT3 induces mitochondrial ROS accumulation in AD, it has been proposed that impaired molecular interactions between SIRT3 and p53 may lead to mitochondrial dysfunction in AD (Hooper *et al*., 2007; Weir *et al*., 2012). In this context, we discovered that that SIRT3 deacetylates p53, thereby reducing p53 occupancy of mitochondrial DNA. In AD, SIRT3 levels are reduced in AD mitochondria leading to increased Ac‐p53 K320 levels and increased p53 occupancy of mitochondrial DNA in AD. Through this mechanism, SIRT3 and p53 contribute to dysregulation of mitochondrial DNA‐encoded gene expression and increased ROS accumulation, and mitochondrial oxygen consumption in AD.

## Results

### SIRT3 mRNA and protein levels are decreased in AD

**Figure 1:**
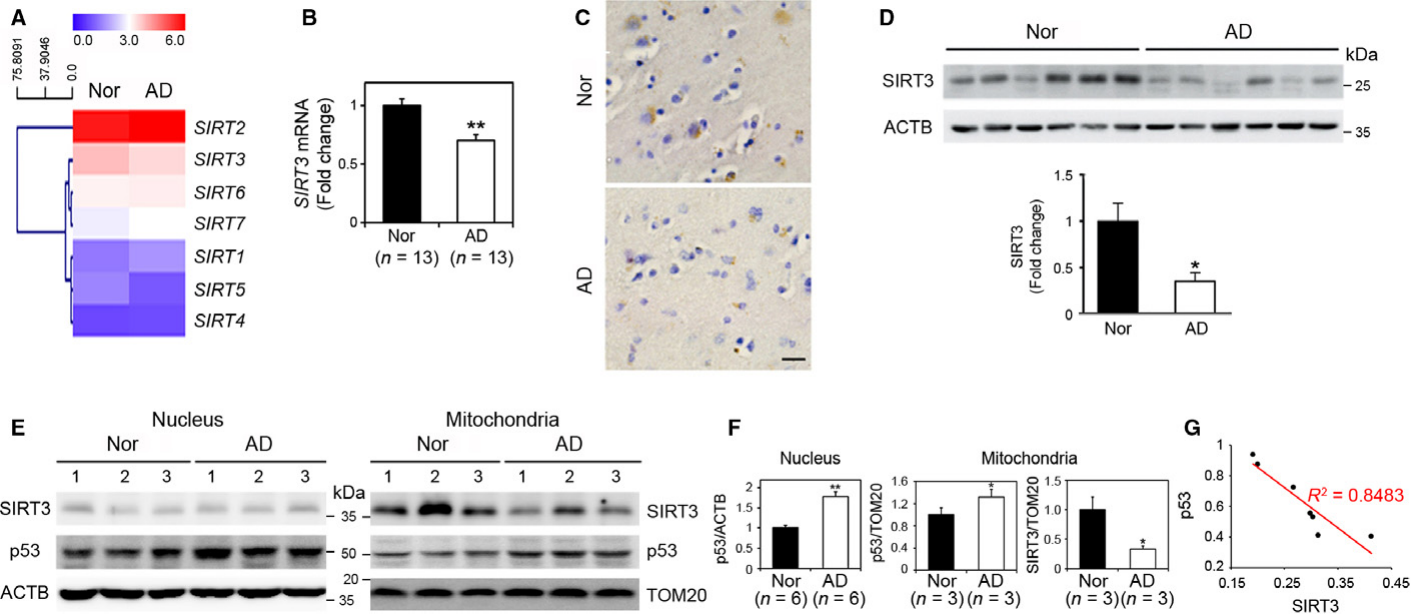
Levels of *SIRT3* mRNA and protein are altered in AD. (A) A heat map showing that basal levels of *SIRT* gene expression are differentially regulated in AD cerebral cortex compared to normal subjects (Nor). Changes in gene expressions are the average of six samples from each group and displayed as higher (red) or lower (purple) color bars. (B) qRT–PCR analysis verified that *SIRT3* mRNA is decreased in AD cortex (*n *= 13) compared to normal subjects (*n *= 13). *GAPDH* was used for normalizing *SIRT3* expression. The bar graph data represents the mean ± SEM (*P <* *0.05). (C) SIRT3 immunoreactivity was decreased in AD frontal cortex. Scale bar: 30 μm. (D) Western blot analysis showed that SIRT3 protein levels are decreased in AD (*n *= 6) compared to controls (*n *= 6). Actin was used as the loading control. Densitometry analysis of SIRT3 protein represented that SIRT3 protein was significantly decreased in AD (*P <* *0.05). (E) Western blot analysis shows that levels of p53 were increased in nuclear and mitochondrial fractions of AD compared to normal subjects, whereas levels of SIRT3 protein were decreased in the mitochondrial fractions in patients with AD. ACTB and TOM20 were used as loading controls of the protein. (F) Densitometry analysis showed a significantly decreased SIRT3 but increased p53 protein levels in patients with AD. The bar graph data represent the mean ± SEM (*P <* *0.05). (G) The regression analysis showed an inverse correlation between SIRT3 and p53 in the mitochondria fractions of patients with AD (*n *= 7, R^2^=0.8438, *P <* *0.05).

In order to identify the SIRT family member (SIRT1 to 7) affected in patients with AD, we first analyzed gene SIRT family expression profiles in the frontal cortex of patients with AD compared to normal subjects. Heat map analysis showed that SIRT3 expression is decreased to a greater degree than other sirtuins in AD (Fig. 1A). SIRT3 was also found to have the second highest level of relative expression among sirtuin family members in brain. Based on these findings, we focused our study on SIRT3 changes associated with AD. Next, to verify the altered SIRT3 gene expression, we examined mRNA expression in frontal cortex of AD and normal subjects using quantitative PCR analysis and found that SIRT3 mRNA is significantly decreased in patients with AD (*n *= 13) compared to normal subjects (*n *= 13) (*P *< 0.05) (Fig. 1B). SIRT3 immunoreactivity was confined to punctate structures within the cytosolic compartment of neurons in the frontal cortex of normal subjects but was markedly reduced and amorphous in patients with AD (Fig. 1C and S6). Western blot analysis confirmed that SIRT3 protein levels are significantly decreased in AD compared to normal controls (*P *< 0.05) (Fig. 1D). Thus, four different methodologies confirmed that SIRT3 mRNA and protein levels are apparently decreased in AD. As it has been shown that p53 expression was increased in AD brain (Hooper *et al*., 2007) and SIRT3 alteration is linked to ROS accumulation in AD (Weir *et al*., 2012), we examined the relationship between SIRT3 and p53 protein levels in AD cortex. p53 protein levels were significantly increased in both nuclear and mitochondrial fractions of patients with AD while SIRT3 protein levels were significantly decreased in the mitochondrial fraction (*P *<* *0.05) (Fig. 1E,F). Linear regression analysis showed an inverse correlation between SIRT3 and p53 protein levels in the mitochondria fraction of patients with AD (Fig. 1G).

### SIRT3 physically interacts with p53

**Figure 2:**
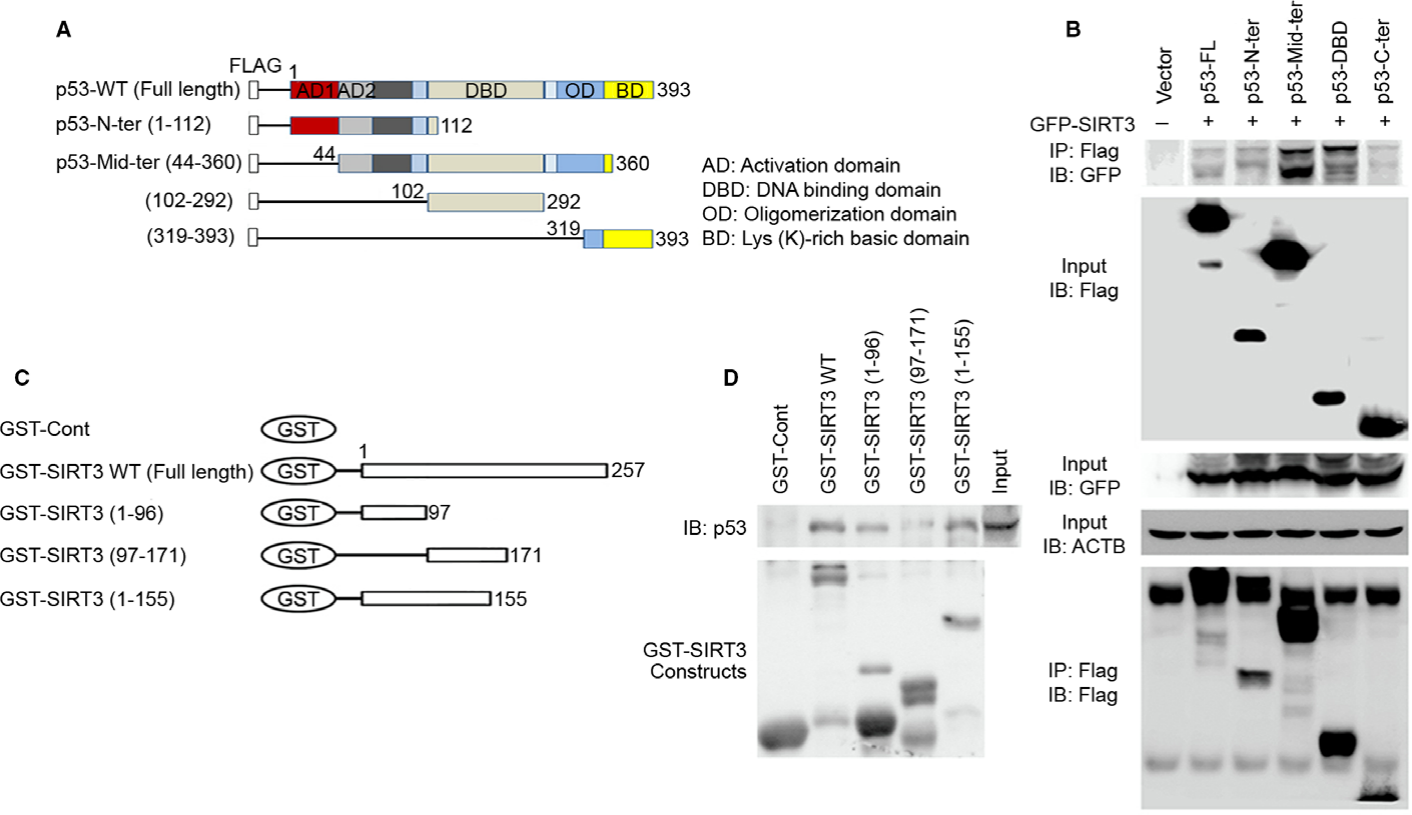
SIRT3 physically interacts with p53. (A) Schematic illustrating full‐length WT p53 and p53 deletion constructs. (B) SIRT3 interacts with the several domains of p53. Flag‐p53 full‐length, Flag‐p53 N‐terminal, Flag‐p53 mid‐terminal, Flag‐p53 C‐terminal, and Flag‐p53 DBD constructs were cotransfected with GFP‐SIRT3 in 293T cells, and equal amounts of protein from cell lysates were immunoprecipitated with anti‐FLAG M2 antibody followed by immunoblotting with anti‐FLAG M2 antibody to detect p53 and with anti‐GFP antibody to detect SIRT3, respectively. A representative experiment is shown. (C) Schematic showing full‐length GST‐SIRT3 and truncated forms of GST‐SIRT3. (D) GST‐SIRT3 pull‐down assay confirmed that SIRT3 is physically associated with p53. *In vitro* GST‐SIRT3 pull‐down was performed with cell lysates extracted from SH‐SY5Y cells with p53 overexpression. GST fusion proteins were visualized by Coomassie blue staining (bottom panel).

**Figure 3:**
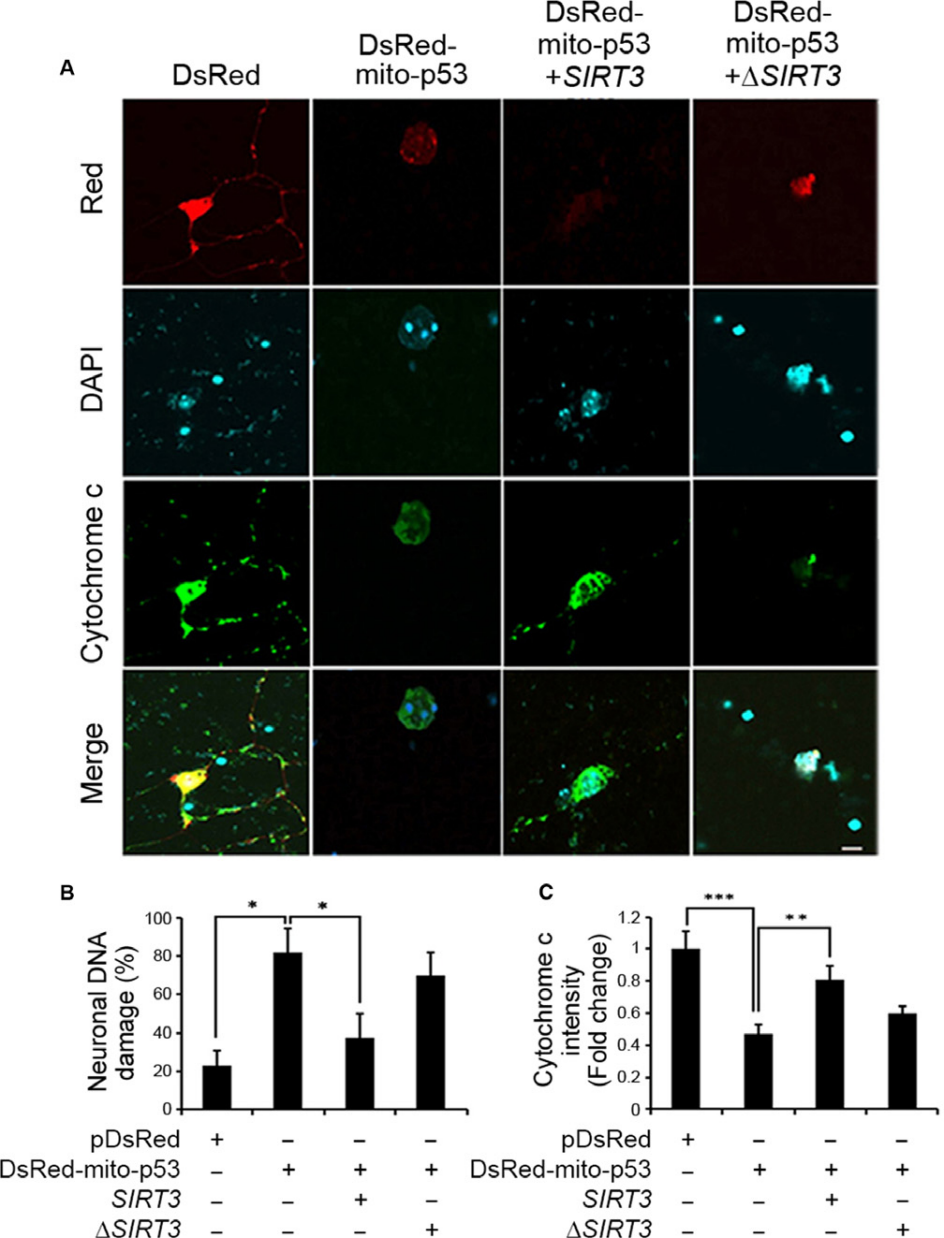
SIRT3 prevents p53‐induced mitochondrial and DNA damage in primary cortical neurons. (A) Photomicrographs show that SIRT3 prevents mito‐p53‐induced neuronal damage. DIV7 primary mouse cortical neurons were transfected with pDsRed control vector or pDsRed mito‐p53 with SIRT3 or ΔSIRT3 for 36 h. Primary neurons were treated with 0.1% saponin (5 min on ice) to release cytosolic content prior to fixation and were subjected to immunostaining for cytochrome c staining and nuclear counterstaining with DAPI. Images were captured by confocal microscopy. Representative images of four separate experiments are shown. Scale bar (white): 10 μm. (B) SIRT3 prevents mito‐p53‐induced neuronal DNA damage. The DNA fragmentation was analyzed from transfected neurons and the bar graphs represent the mean ± SEM (*n *= 4; *P <* *0.05). (C) SIRT3 prevents mito‐p53‐induced mitochondrial damage. The bar graphs represent relative endogenous cytochrome c (FITC) intensity that is quantified using Nikon NIS‐Elements AR software. Error bars represent S.E.M (*n *= 4; **P <* *0.01, ***P <* *0.001).

To examine molecular interactions between SIRT3 and p53, we used several constructs including as Flag‐p53 full‐length, Flag‐p53 N‐terminal, Flag‐p53 mid‐terminal, Flag‐p53‐DBD, and Flag C‐terminal in *in vitro* associations assays (Fig. 2A). The above p53 constructs were cotransfected with GFP‐SIRT3 in 293T cells, and SIRT3 protein was immunoprecipitated by anti‐GFP antibody. As shown in Fig. 3B, p53 mid‐terminal region strongly interacted with SIRT3. To further confirm the region or domain of SIRT3 interacting with p53*,* we performed a GST pull‐down assay using wild‐type GST‐SIRT3 and three GST‐SIRT3 truncated fragments (Fig. 2C). Our results showed that the N‐terminal of SIRT3 protein preferentially interacts with p53 (Fig. 2D).

### SIRT3 prevents mitochondrially targeted p53 (mito‐p53)‐induced neuronal damage and mitochondrial dysfunction by deacetylating p53 at K320

To define the effects of SIRT3 on p53‐dependent neuronal activity, we cotransfected mito‐p53 with WT SIRT3 or ΔSIRT3 in DIV7 primary cortical neurons and determined neuronal DNA damage and mitochondrial function (Onyango *et al*., 2002; Shi *et al*., 2005). As expected, ectopic expression of mito‐p53 induced significant (around 80%) neuronal DNA damage (indicating DNA fragmentation by DAPI staining) compared to control vector (DsRed)‐transfected neurons (around 20%) (Fig. 3A,B). WT SIRT3 overexpression significantly decreased mito‐p53‐induced DNA damage to around 40%, whereas mutant ΔSirt3 did not significantly prevent mito‐p53‐induced DNA damage (around 70%) (*P *<* *0.05) (Fig. 3A,B). We assayed the cytochrome c levels using immunofluorescence staining as a measure of mitochondrial dysfunction (Mihara *et al*., 2003). Mito‐p53‐transfected neurons showed two times lower intensity than control neurons (control vector‐transfected cells). WT SIRT3 overexpression recovered more than 80% of the cytochrome c intensity to a level similar to control neurons while mutant ΔSIRT3 did not restore cytochrome c intensity (*P *<* *0.05) (Fig. 3A,C). Together, our data indicate that SIRT3 plays a protective role against mito‐p53‐induced DNA damage and mitochondrial dysfunction in primary neurons.

**Figure 4:**
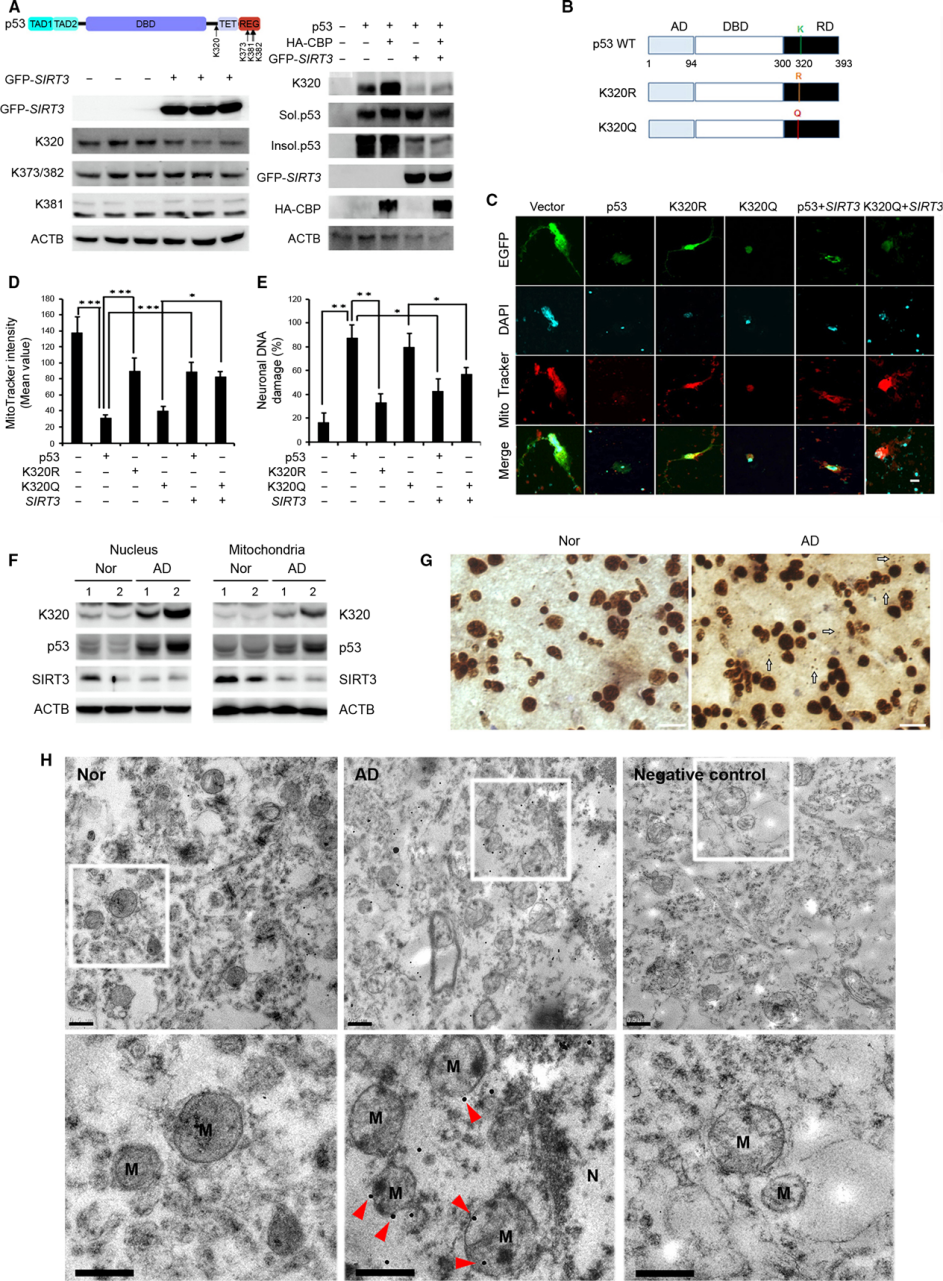
SIRT3 regulates deacetylation of p53 at K320 and p53‐induced neuronal damage. (A) SIRT3 deacetylates p53 at K320. H1299 p53 KO cells were cotransfected p53 with HA‐CBP, or with GFP‐SIRT3 for 24 h, respectively. ACTB was used as loading control. (B) Schematic structures show constructs of wild‐type p53, K320R mutant, and K320Q mutant. (C) p53 induces neuronal damage in an acetylation of K320‐dependent pathway. DIV7 primary mouse cortical neurons were cotransfected EGFP with vector, p53, K320R, K320Q, p53 and SIRT3, and K320Q and SIRT3 for 36 h. Neurons were stained with MitoTracker Red CMXRos for 30 min prior to fixation and were subjected in DAPI staining. Scale bar (white): 10 μm. (D) SIRT3 modulates mitochondrial activity through the deacetylation of p53 K320. The graph data represent the mean ± SEM (*n *= 4; *P < 0.05, ***P < 0.001). (E) SIRT3 modulates p53‐induced DNA damage through the deacetylation of p53 K320. The DNA fragmentation was analyzed from transfected neurons and the bar graphs represent the mean ± SEM (*n *= 4; *P < 0.05, **P < 0.01). (F) Western blot analysis shows that levels of acetylated p53 at K320 were increased in both nucleus and mitochondria fractions of AD compared to control, whereas SIRT3 was decreased in mitochondria fractions of AD. ACTB and COX4 were used as the loading control. (G) Immunoreactivity of acetylated p53 at K320 was increased in the nucleus and the mitochondria of AD. Arrows (white) indicate cytoplasmic and mitochondrial localization of acetylated p53K320 signals. Scale bar (white): 20 μm. (H) Electron micrograph showed that immunogold‐labeled particles of acetylated p53 at K320 are found in the mitochondria of cortical neurons and its level is increased in patient with AD compared to normal (Nor) subject. Arrowheads (red) indicate cytoplasmic and mitochondrial localization of acetylated p53K320 signals. M, mitochondria; N, nucleus. Scale bars: 500 nm.

Several studies have shown the apoptotic role of p53 acetylation at the K320 residue (Sakaguchi *et al*., 1998; Terui *et al*., 2003; Roy *et al*., 2005; Brandl *et al*., 2012). In order to determine whether SIRT3 modulates p53 acetylation, we performed Western blot analysis after cotransfection of SIRT3 and p53 constructs in SH‐SY5Y cells. SIRT3 overexpression reduced p53 acetylation at K320, but not at other lysine residues including K373, K381, and K382 in SH‐SY5Y cells (Fig. 4A, left panel). To further verify the role of SIRT3 in p53 acetylation at K320, we cotransfected CBP/p300 which is generally known to induce p53 acetylation (Ito *et al*., 2001; Koh *et al*., 2014) and ran Western blot analysis. As shown in Fig. 4A (right panel), while CBP robustly increased p53 acetylation at K320, SIRT3 effectively reduced p53 acetylation at K320 by CBP. To further validate whether p53 acetylation at K320 is associated with neuronal damage, primary neurons were transfected with an acetylation site mutant p53 (K320R) and an acetylation mimetic mutant p53 (K320Q) and stained with DAPI and MitoTracker (Fig. 4B,C). MitoTracker intensity is a well‐established surrogate for mitochondrial membrane potential (MMP) (Favret & Lynn, 2010). Our data showed that MitoTracker intensity was significantly decreased in both WT p53‐transfected and mutant p53 K320Q‐transfected neurons compared to control or mutant p53 K320R‐transfected neurons (*P *<* *0.05) (Fig. 4B–D). Notably, WT SIRT3 recovered WT p53‐ or mutant p53K320Q‐induced mitochondrial membrane permeability transition (MMPT) (*P *<* *0.05) (Fig. 4C,D). In addition, WT SIRT3 prevented WT p53‐ or mutant p53K320Q‐induced neuronal DNA fragmentation (*P *<* *0.05) (Fig. 4C,E). To confirm whether acetylated p53K320 level is altered in AD, we measured acetylated p53 at K320 using Western blot analysis, immunohistochemistry, and immunogold labeling and TEM analysis in AD and control frontal cortex. The level of acetylated p53 at K320 was significantly increased in patients with AD (*n *= 6) compared to normal subjects (*n *= 6) (Fig. 4F,G). The gold particles for acetylated p53K320 were found in the mitochondria and p53K320‐positive gold particles were increased in patients with AD compared to normal subjects (Fig. 4H). Accordingly, our results demonstrate that both the WT p53 and acetylated p53 at K320 are altered by AD pathology.

**Figure 5:**
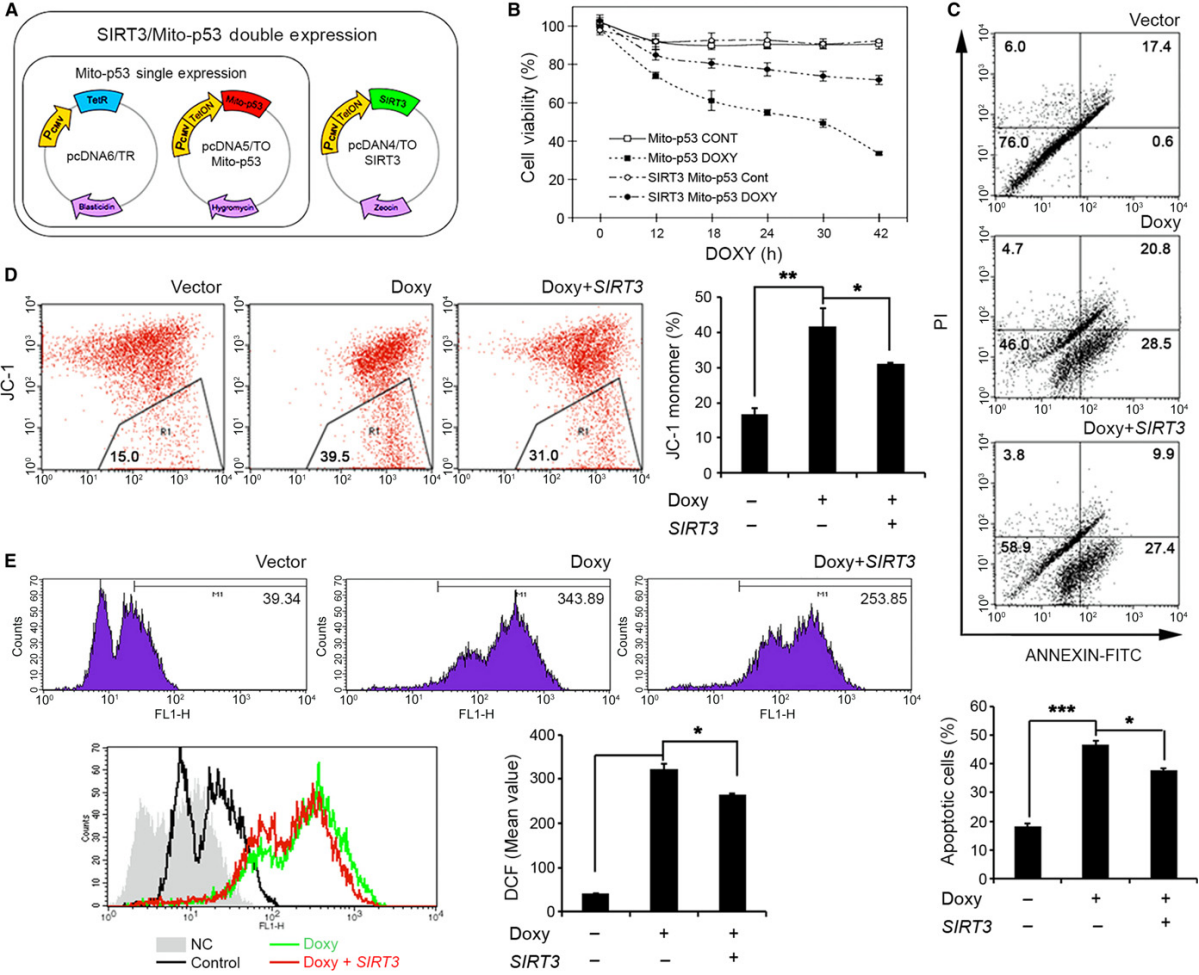
SIRT3 prevents mito‐p53‐induced mitochondrial dysfunction and neuronal death. (A) A scheme shows SIRT3/mito‐p53 double expression cell model. (B) SIRT3 improved mito‐p53‐induced cell viability. SIRT3/mito‐p53 double or mito‐p53 single inducible cell lines were treated with or without 2 μg mL^−1^ DOXY for 0, 12, 18, 24, 30, and 42 h, respectively. Cell viability was analyzed by MTT assay. (C) Ectopic expression of SIRT3 prevented mito‐p53‐induced apoptosis. Apoptotic cells were analyzed by flow cytometry after Annexin V/ PI staining in mito‐p53‐inducible cell with or without SIRT3 overexpression. The bar graph data (bottom panel) represents the mean ± SEM (*n *= 3; *P <* *0.05, ***P <* *0.001). (D) SIRT3 improved mito‐p53‐induced mitochondrial membrane potential (Ψ) loss. Mitochondrial membrane potential was detected by flow cytometry after JC‐1 staining in mito‐p53‐inducible cell with or without SIRT3 overexpression. The bar graph data represent the mean ± SEM (*n *= 3; *P <* *0.05, **P <* *0.01). (E) Ectopic expression of SIRT3 reduced mito‐p53‐induced ROS production. Reactive oxygen species (ROS) were analyzed by flow cytometry after DCF staining in mito‐p53‐inducible cell with or without SIRT3 overexpression. The bar graph data represent the mean ± SEM (*n *= 3; *P <* *0.05).

### SIRT3 prevents mito‐p53‐induced cell death and mitochondrial dysfunction

To gain more insight into the role of SIRT3 in mito‐p53‐induced cell death and mitochondrial dysfunction, tetracycline‐inducible mito‐p53 SH‐SY5Y cell lines were developed. Figure 5A depicts the strategy we used to generate inducible mito‐p53 and SIRT3 cell lines. Doxycycline (DOXY) was applied for the indicated time periods, and the cell viability was measured. When mito‐p53 was induced by DOXY treatment, cell viability was markedly and progressively decreased over time. In contrast, induction of SIRT3 together with mito‐p53 significantly prevented cell death (*P *<* *0.05) (Figs 5B and S1A,B, Supporting information). Previous reports have shown that SIRT3 plays a protective role in diverse stress‐induced cell death (Hagen *et al*., 1995; Kim *et al*., 2007; Hirschey *et al*., 2010). To determine whether SIRT3 prevents mito‐p53‐induced toxicity, mito‐p53 cells were transfected with a control vector, WT SIRT3, or mutant ΔSIRT3 and cells were then stained with Annexin V and propidium iodide (PI). WT SIRT3 overexpression, but not mutant ΔSIRT3, significantly decreased mito‐p53‐induced early and late apoptosis (*P *<* *0.05) (Figs 5C and S1C). Our data strongly indicate that WT SIRT3 rescues mito‐p53‐dependent late apoptosis. Next, in order to detect MMP changes, we stained mito‐p53 cells in the presence of WT SIRT3 or mutant ΔSIRT3 with JC‐1. JC‐1 monomer was significantly increased in DOXY‐treated mito‐p53 cells (R1 area of flow cytometry scatter plot). WT SIRT3 overexpression significantly decreased the formation of JC‐1 monomer in mito‐p53 cells (*P *<* *0.05) (Fig. 5D). Mutant ΔSIRT3 did not rescue MMP levels reduced by mito‐p53 (Fig. S1D). Normal SH‐SY5Y cells were transfected with WT p53, mutant p53K320Q, or mutant p53K320R in the presence and absence of WT SIRT3 followed by Annexin V/PI staining. We found that WT SIRT3 rescues WT p53‐ and mutant p53K320Q‐induced cell death (*P *<* *0.05) (Fig. S2). We then performed DCF‐DA staining and flow cytometry analysis to determine whether oxidative stress pathway is associated with mito‐p53‐dependent mitochondrial dysfunction. ROS accumulation was robustly increased in DOXY‐treated mito‐p53 cells (Fig. 5E). WT SIRT3 overexpression significantly decreased ROS accumulation in DOXY‐treated mito‐p53 cells (*P *<* *0.05) (Fig. 5E). Our results demonstrate that SIRT3 plays a protective role against mito‐p53‐induced ROS‐mediated mitochondrial dysfunction and cell death (Fig. S8).

### SIRT3 regulates p53‐dependent mitochondrial genome expression and modulates mitochondrial ROS level and oxygen consumption

**Figure 6:**
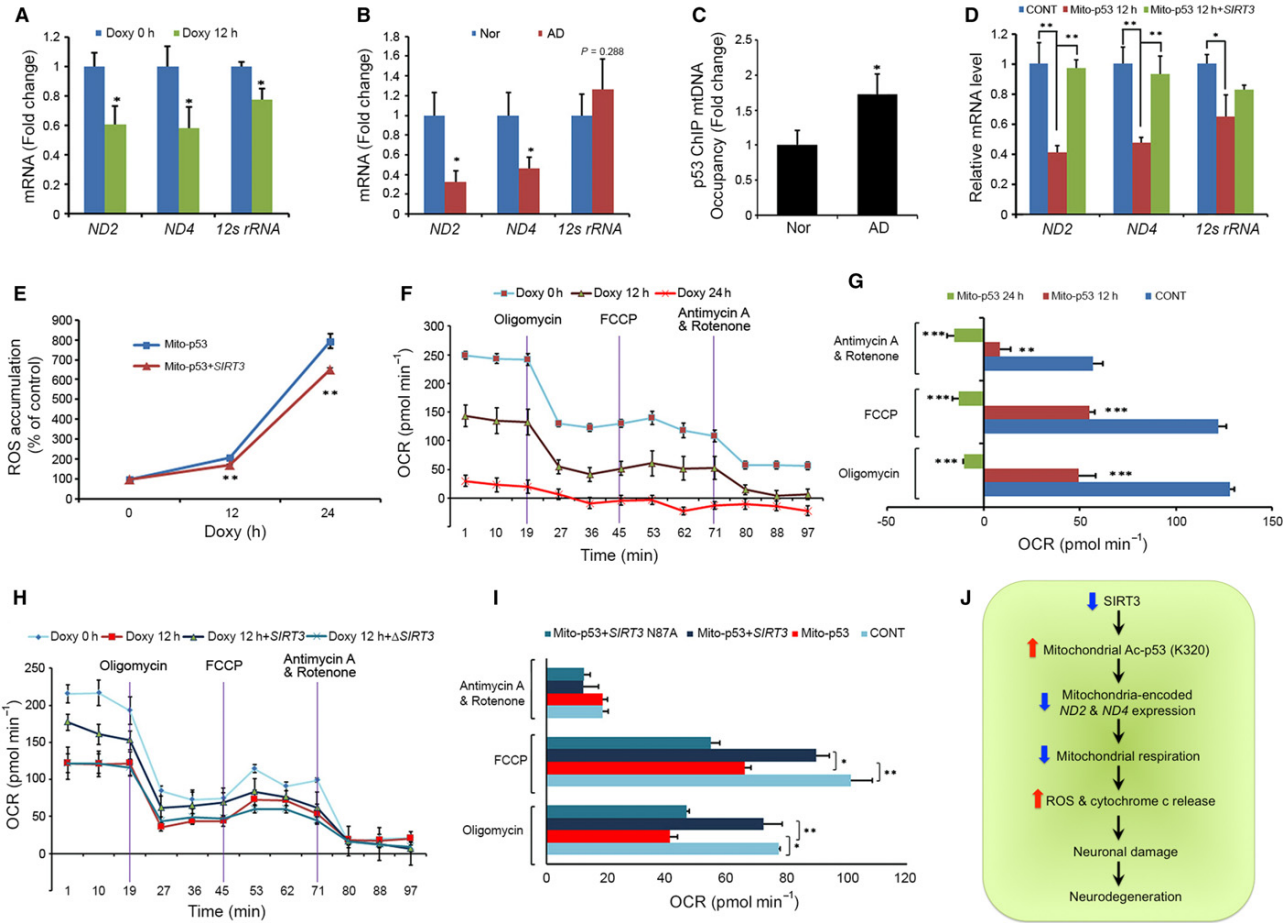
SIRT3 involves in mito‐p53‐dependent mitochondrial genome expression, mitochondrial ROS level, and oxygen consumption. (A) Mito‐p53 repressed mitochondria‐encoded gene expression. The bard graph data represent the mean ± SEM (*n *= 3; *P < 0.05). (B) The mRNA levels of ND2 and ND4 were significantly reduced in the cortex of patients with AD compared to normal subjects. The graph data represent the mean ± SEM (*n *= 6; *P < 0.05). (C) Quantitative ChIP analysis confirmed that p53 occupancy to mitochondrial DNA was elevated in the cortex of patients with AD compared to normal subjects (*n *= 6; *P < 0.05). (D) SIRT3 rescued expression of mitochondria‐encoded genes that are repressed by mito‐p53. The bar graph data represent the mean ± SEM (n = 3; *P < 0.05, **P < 0.01). (E) SIRT3 reduced mito‐p53‐induced oxidative stress (ROS accumulation). Cells were subjected to DCF‐DA staining followed by flow cytometry. The line graph data represent the mean ± SEM (*n *= 5; **P < 0.01). (F) Mito‐p53 reduced mitochondrial oxygen consumption and respiratory function. Cells were applied to oligomycin, FCCP, antimycin, and rotenone followed by oxygen consumption rate (OCR) analysis. (G) The OCR level was significantly reduced by mito‐p53. The bar graphs were originated from (E) panel (*n *= 5, **P < 0.01, ***P < 0.005). (H) SIRT3 rescues mitochondrial oxygen consumption and respiratory function affected by mito‐p53. (I) SIRT3 significantly restores the OCR level decreased by mito‐p53. The bar graphs were originated from (H) panel (*n *= 5, *P < 0.05, **P < 0.01). (J) A flowchart illustrates that, in a condition of AD pathology, a reduction in SIRT3 levels leads to an increase in acetylated p53 (K320) levels in the mitochondria and impairs mito‐p53‐dependent mitochondrial genome expression, resulting in elevations of ROS accumulation and neuronal damage.

To further investigate whether mitochondrially targeted p53 directly regulates mitochondrial genome expression and SIRT3 modulates p53‐dependent mitochondrial gene expression, we performed a series of experiments including ChIP and qRT–PCR. As shown in Fig. 6A, induction of mito‐p53 by DOXY treatment significantly decreased expression of *ND2*,* ND4,* and *12S rRNA* genes at 12 h (*P *<* *0.05). Other mitochondria genome‐encoded genes were partially affected by mito‐p53 induction (Fig. S3). Consistent with the result of DOXY‐inducible mito‐p53 cells, both *ND2* and *ND4* mRNA levels were significantly decreased in the frontal cortex of patients with AD compared to normal subjects while there was no significant change in the mRNA level of *12S rRNA* (*P *<* *0.05) (Fig. 6B and S7). Next, we applied p53‐chromatin immunoprecipitation (ChIP) and qPCR assays to identify whether p53 occupies mitochondrial DNA (mtDNA) and whether its occupancy is altered in AD. Interestingly, p53 occupancy of mtDNA was markedly increased in patients with AD compared to normal subjects (*P *<* *0.05) (Fig. 6C). Additionally, DNA sequencing of p53‐ChIP qPCR product verified that p53 preferentially occupies the *ND2* and *12S rRNA* mitochondrial genes (Fig. S4). To test whether putative mitochondrial consensus p53‐binding element (mito‐p53 BE) is transcriptionally regulated by p53 (Heyne *et al*., 2004), we generated mito‐p53 BE‐driven reporter construct using pGL3E luciferase vector (Fig. S5A). p53 induced mito‐p53 BE‐driven reporter activity in a dose‐dependent manner, indicating that p53 can directly participate in transcriptional regulation of the mitochondrial genome (Fig. S5B,C). WT SIRT3 decreased mito‐p53 BE‐driven reporter activity (Fig. S5D). These data suggest that SIRT3 modulates p53‐dependent mitochondrial transcriptional activity. Even though the reporter assay was designed for testing participation of p53 in mitochondrial genome transcription via mito‐p53 BEs, mito‐p53 BE‐driven reporter activity did not exactly correlate with net levels of mitochondrial gene expression in cell lines and human AD brains.

WT SIRT3 rescued *ND2* and *ND4* mRNA levels in mito‐p53 cells, indicating that WT SIRT3 modulates mitochondrial *ND2* and *ND4* expression that is negatively regulated by mito‐p53. (*P *<* *0.05) (Fig. 6D). Because mito‐p53 may affect intracellular ROS levels via repression of mitochondria‐encoded Complex I genes such as *ND2* and *ND4*, we measured alterations in ROS level and oxygen consumption rate (OCR) in DOXY‐treated mito‐p53 cells. Mito‐p53 induction increased ROS levels and reduced oxygen consumption capacity in a time‐dependent manner (*P *<* *0.05) (Fig. 6E–G). WT SIRT3 decreased ROS levels and increased OCR in DOXY‐treated mito‐p53 cells (*P *<* *0.05) (Fig. 6E,H,I). Mutant ΔSIRT3, however, did not reduce ROS levels or increase OCR in DOXY‐treated mito‐p53 cells (Fig. 6G,H). These data suggest that mito‐p53‐induced Complex I‐dependent mitochondrial dysfunction causes ROS accumulation by reducing *ND2* and *ND4* mRNA levels. SIRT3 restores mito‐p53‐reduced basal mitochondrial oxygen consumption and mitochondrial oxidative capacity by rescuing mito‐p53‐reduced *ND2* and *ND4* mRNA levels and reducing mito‐p53‐changed ROS levels. To further verify whether ND2 and ND4 mediate a neuroprotective effect in response to SIRT3 and mito‐p53, loss of function of *ND2* and *ND4* was analyzed using siRNAs as shown in Fig. S9 (Table S3). Knock down of *ND2* and *ND4* by siRNAs significantly decreased SIRT3‐dependent cell viability in SH‐SY5Y cells. On the other hand, knock down of *ND2* and *ND4* by siRNAs increased levels of cleaved caspase‐3 in SH‐SY5Y cells.

## Discussion

### Alterations of SIRT3 and mitochondrial p53 levels are associated with AD pathology

Mitochondrial dysfunction has been closely linked to the pathogenesis of AD, but the relationship between mitochondrial pathology and neuronal damage is poorly understood (Bonilla *et al*., 1999; Swerdlow, 2012). In the present study, we found that reduced mitochondrial SIRT3 is associated with altered mitochondrial respiratory activity and neuronal damage in AD. SIRT3 mRNA and mitochondrial SIRT3 protein levels were significantly decreased, and mitochondrial p53 protein was significantly increased in AD cortex. Furthermore, levels of mitochondrial SIRT3 are inversely correlated with mitochondrial p53 in AD cortex, suggesting that mitochondrial SIRT3 and p53 alterations may contribute to AD pathogenesis. A significant downregulation of SIRT3 among SIRT family members indicates that SIRT3 expression may be regulated in a gene context‐dependent manner in the context of AD. We propose that the disparity between SIRT3 mRNA and protein levels in AD may be due to a differential sensitivity of assay methods between qPCR vs. Western blot. Otherwise, it is possible that differential alteration of transcription vs. translation may contribute to the different levels of SIRT3 mRNA and protein through a yet unknown pathway.

### SIRT3 deacetylates p53 at K320 and prevents p53 neurotoxicity

The immunoprecipitation data showed that SIRT3 interacts with middle regions of the p53 protein (amino acids 44‐360). GST pull‐down experiments confirmed that the N‐terminal of SIRT3 (amino acids 1‐97) interacts with several p53 domains including both activation and DNA binding domains. Our data are consistent with a previous report that SIRT3 interacts with the mitochondria‐associated senescence domain (MASD) between amino acids 64 and 209 of p53 (Li *et al*., 2010). Interestingly, it has shown that SIRT3 rescues p53‐induced growth arrest and senescence and Bcl2‐associated athanogene 2 (BAG‐2), a chaperone protein, inhibited the rescue function of SIRT3 (Li *et al*., 2010). The coordinated interaction between p53, SIRT3, and BAG‐2 is closely linked to the regulation of cell fate through a transcription‐independent pathway (Li *et al*., 2010).

Most notably, for the first time, we identified that SIRT3 specifically reduces the level of Ac‐p53 K320 and further blocks CBP‐induced p53 acetylation at K320. These data suggest a *bona fide* role for SIRT3 as a deacetylase that catalyzes the deacetylation of p53 at K320. To further determine how Ac‐p53 K320 affects neuronal activity, we examined the function of mutant p53 K320R (a mimic of non‐acetyl lysine) and K320Q (a mimic of acetyl lysine) in primary neurons. As expected, the p53 K320Q mutant induced mitochondrial dysfunction and cell death, while the p53 K320R mutant did not. In addition, SIRT3 overexpression nullified the pathological changes induced by WT p53 and p53 K320Q, supporting our hypothesis that SIRT3 prevents p53‐induced mitochondrial dysfunction and neuronal cell death. Previous studies support our finding that p53 acetylation at the K320 residue is linked to cell damage (Sakaguchi *et al*., 1998; Terui *et al*., 2003; Roy *et al*., 2005; Brandl *et al*., 2012). Importantly, we found that Ac‐p53 K320 levels are significantly increased in the mitochondria of AD in conjunction with reduced SIRT3 levels. In this context, failure of deacetylation of mitochondrial Ac‐p53 at K320 due to the decreased SIRT3 activity may initiate cascades of mitochondria‐dependent neuronal damage processes in AD.

### SIRT3 modulates p53‐dependent mitochondrial genome expression and mitochondrial activity

We determined that SIRT3 modulates mito‐p53‐dependent mitochondrial gene expression and mitochondrial respiratory function. As a result, SIRT3 prevented mito‐p53‐induced mitochondrial dysfunction and neuronal damage in both primary neurons and SH‐SY5Y cells. Mitochondrial *ND2* and *ND4* genes encode core subunits of the mitochondrial membrane respiratory chain NADH dehydrogenase (Complex I) (Mathiesen & Hagerhall, 2002; Brandt, 2006). Complex I functions in the transfer of electrons from NADH to the respiratory chain (Hayashi & Stuchebrukhov, 2010). Previous studies have shown that mRNA levels of mitochondrial genome‐encoded Complex I (*ND4*), *COX I, COX II,* and *COX III* genes are significantly decreased in AD temporal cortex (Chandrasekaran *et al*., 1996; Fukuyama *et al*., 1996). Our results suggest that reduced SIRT3 in AD causes mito‐p53‐dependent repression of *ND2*,* ND4,* and *12s rRNA* gene expression.

The association between p53 and mtDNA had been hypothesized because of its localization in the mitochondrial inner matrix (Fukuyama *et al*., 1996). We identified that p53 binds to potential consensus binding elements in the human mitochondrial genome and p53 binding to mtDNA was elevated in AD. It seems likely that the higher p53 occupancy to mtDNA is related to repression of *ND2* and *ND4* gene expression in AD. In this context, SIRT3 may modulate p53 binding to mtDNA by deacetylating p53 at K320 and thereby coordinate mitochondrial gene expression. We previously reported that cAMP response element binding protein (CREB) is localized to neuronal mitochondria and specifically binds to CREB response elements in the mitochondrial genome that regulate mitochondrial‐encoded Complex I gene expression (Lee *et al*., 2005; Ryu *et al*., 2005). In the current study, we provide another layer of mechanism that increased mito‐p53 level leads to ROS accumulation and reduces the rate of mitochondrial oxygen consumption. In contrast, SIRT3 prevents ROS accumulation and restores mitochondrial oxygen consumption by modulating mito‐p53 activity. Collectively, our data indicate that the deregulation of SIRT3 impairs mitochondrial gene expression via mitochondrial p53 activation in AD. Previous studies have shown that mitochondrial ROS are generated both via mitochondrial respiration and by oxygen‐independent processes under physiologic conditions in a cell type‐specific and metabolic rate‐dependent manner (Kushnareva *et al*., 2002; Barja, 2007). Based on previous findings, we expect that the increased ROS levels and concomitant reduced oxygen consumption capacity caused by mito‐p53 may contribute to neuronal dysfunction. How ROS generation is regulated independent of oxygen consumption warrants further investigator in the future.

### A novel role of p53 in the mitochondria of AD

Since their initial finding of mitochondrial p53 in 2000 (Marchenko *et al*., 2000), Moll and colleagues have performed pioneering studies, showing that p53 is specifically targeted to mitochondria and plays transcription‐independent pro‐apoptotic functions (Mihara *et al*., 2003; Erster & Moll, 2004; Moll *et al*., 2005). p53 directly interacts with multidomain proteins of the Bcl‐2 family at the mitochondrial outer membrane and induces mitochondrial outer membrane permeabilization, which is a prominent apoptotic checkpoint (Mihara *et al*., 2003; Tomita *et al*., 2006; Follis *et al*., 2014). Several stresses such as hypoxia and ischemia induce translocation of p53 protein to mitochondria (Sansome *et al*., 2001; Erster & Moll, 2004; Vaseva *et al*., 2012). Mitochondrially targeted p53 leads to cell death in solid tumor models (Palacios *et al*., 2008). Intriguingly, p53 physically interacts with cyclophilin D (CypD) and the p53‐CypD complex results in mitochondrial permeability transition pore opening and mediates cell death caused by ischemic injury. Cyclosporine A pretreatment or reduction of p53 proteins prevents formation of the p53‐CypD complex in an ischemic injury mouse model (Vaseva *et al*., 2012). In the current study, we found that SIRT3 rescues mito‐p53‐induced cytochrome c release in primary neurons. Release of cytochrome c from mitochondria is a well‐known upstream event to trigger caspase‐3‐dependent neural cell damage. Teng *et al*. (2004) have shown that spinal cord injury (SCI) increases mitochondrial cytochrome c release and minocycline treatment provides neuroprotective effect by reducing cytochrome c release in SCI. These data suggest that therapeutic regulation of mitochondrial function may be an effective approach to treat neurotrauma. Whether this therapeutic approach modulates SIRT3 and mito‐p53‐dependent pathway remains to be investigated.

The novel roles of p53 in the mitochondria have been well documented (Park *et al*., 2016). p53 interacts with mtDNA‐associated proteins such as CHCHD4, OGG1, Parkin, POLG, and TFAM (Achanta & Huang, 2004; Achanta *et al*., 2005; and Wong *et al*., 2009; Hoshino *et al*., 2013; Zhuang *et al*., 2013). In conjunction with partner molecules, p53 is involved in modulating mtDNA replication and transcription, maintaining mtDNA stability, and repairing mtDNA. Bergeaud *et al*. recently showed that mitochondrial p53 interacts with oligomycin sensitivity‐conferring protein, a component of F1F0‐ATP synthase complex, and promotes the assembly of F1F0‐ATP synthase complex (Bergeaud *et al*., 2013). These reports show that p53 is directly involved in mitochondrial respiratory function through its interaction with mitochondrial matrix proteins. Our novel findings show that elevation of mitochondrial p53 activity contributes to the pathogenesis of AD through its role as a mitochondrial transcription factor. The mitochondrial p53 activity was modulated by SIRT3 in neurons. Based on our current study, further work will be necessary to define what signals and other factors are associated with p53‐mediated mitochondrial transcription normally and in neurodegenerative conditions such of AD.

In summary, we found reduced SIRT3 levels in neuronal mitochondria in AD that inversely correlate with increased levels of mitochondrial p53. In addition, levels of Ac‐p53 K320 were elevated in neuronal mitochondria of AD. Decreased SIRT3 levels lead to mitochondrial dysfunction via p53‐mediated reduction of mitochondria genome‐encoded *ND2* and *ND4* expression in AD. Consequently, altered activity of SIRT3 and p53 impaired mitochondrial oxygen consumption and enhanced neuronal damage in AD. Taken together, alterations of SIRT3 and mitochondrial p53 may be a marker for mitochondrial dysfunction in AD and therapeutic modulation of SIRT3 activity may be a useful strategy to ameliorate mitochondrial pathology in AD.

## Experimental procedures

### Cell culture

Mouse cortical primary neurons from P0 pups were cultured in Neurobasal medium supplemented with 1XB‐27 supplement and 1X L‐glutamine (Invitrogen, CA, USA) in a humidified atmosphere of 5% CO2 at 37°C. 293T cells, SH‐SY5Y cells, and doxycycline (DOXY)‐inducible mitochondrially targeting p53 (mito‐p53) cells were cultured in Dulbecco's modified Eagle's medium (DMEM; Thermo Scientific, Waltham, MA, USA) supplemented with 10% fetal bovine serum) and 1% penicillin/streptomycin (P/S) in a humidified atmosphere of 5% CO2 at 37°C.

### Chemicals and antibodies

Anti‐SIRT3, anti‐COX4, anti‐FLAG M2, and anti‐ACTB antibodies were obtained from Sigma (St. Louis, MO, USA). Rabbit anti‐HA and mouse anti‐p53 (DO‐1) antibodies were purchased from Santa Cruz (Dallas, TX, USA). Rabbit anti‐acetyl‐p53 K373/382 and rabbit anti‐acetyl‐p53 K320 antibodies were purchased from Millipore (Billerica, MA, USA). Rabbit acetyl‐p53 K381 antibody was purchased from Abcam (Cambridge, MA, USA). Mouse anti‐cytochrome c antibody (Clone: 6H2.B4) was purchased from BD Pharmingen (Franklin Lakes, NJ, USA). Secondary antibodies including goat anti‐mouse IgG and goat anti‐rabbit IgG were purchased from Jackson ImmunoResearch (West Grove, PA, USA).

### Human brain samples

Neuropathological processing of control and AD human brain samples was performed using procedures previously established by the Boston University Alzheimer's Disease Center (BUADC). All brains were donated with consent of the next of kin after death. Institutional review board approval was obtained through the BUADC. The study was performed in accordance with institutional regulatory guidelines and principles of human subject protection in the Declaration of Helsinki. Detailed information of brain tissues is described in Table S1.

### Quantitative real‐time PCR (qPCR)

Total RNA was isolated from brain tissues using a commercial extraction system (Qiagen). 1 μg total RNA has been used for cDNA preparation with iScript cDNA Synthesis Kit (Bio‐Rad, Hercules, CA, USA) according to the manufacturer's protocols. cDNA from each sample was amplified by real‐time PCR using iQ SYBR Green Supermix (Bio‐Rad). RNA quantities were normalized using GAPDH mRNA as a reference. PCR cycling conditions were as follows: denaturation for 3 min at 95°C; then 40 cycles of amplification for 15 s at 95°C, 15 s at 60°C, 20 s at 70°C; followed by 30 s at 72°C. For melt curve, data collection has been used 33 cycles, 6 s each, with the temperature increased from 60°C to 92°C (increase set point temperature after cycle 2 by 1°C). The PCR primer for SIRT3 was as follows: forward, 5’‐CGGCTCTACACGCAGAACATC‐3’ and reverse, 5’‐CAGCGGCTCCCCAAAGAACAC‐3’; the PCR primer for ND2 was as follows: forward, 5’‐ GAGTAGATTAGGCGTAGGTA‐3’ and reverse, 5’‐ CGGCCTGCTTCTTCTCA‐3’; ND4: forward, 5’‐ CTAGGCCATATGTGTTGGA‐3’ and reverse, 5’‐ GTATATCGCCTCACACCTCA‐3’; and 12s rRNA: forward, 5’‐ CGGTATATAGGCTGAGCA‐3’ and reverse, 5’‐ GGAACAAGCATCAAGCA‐3’ (Table S2).

### Immunogold labeling and transmission electron microscopy (TEM)

Immunogold labeling and TEM were performed with some modification as previously described (Lee *et al*., 2005; Ryu *et al*., 2005). The tissues were fixed with 2.5% glutaraldehyde dissolved in 0.1M cacodylate buffer overnight at 4°C and with 2% osmium tetroxide for 1 h. The cells were dehydrated with ethanol series, infiltrated with Spurr's resin series, and polymerized at 60°C for 8 h. The embedded cell was cut with a diamond knife on ultramicrotome (Ultracut S, Leica). The sections were mounted directly on 150‐mesh nickel grids. The sections were treated with 3% H_2_O_2_ for 30 min and incubated with the AURION Blocking Agent for 30 min. Then, the sections were incubated with the anti‐acetylated‐p53 (Ac‐p53)‐rabbit IgG. The sections were then rinsed with 0.2%‐BSA‐C (AURION, the Netherlands) in PBS and incubated with 30‐nm gold‐conjugated anti‐rabbit antibody for 1 h. After washing with 0.1% BSA‐C in PBS, the sections were treated with 0.2% glutaraldehyde and washed with distilled water. The sections were stained with 2% uranyl acetate solution for 10 min. The grids were examined with Tecnai F20 (FEI, the Netherlands) at 200 kV. Negative control was incubated with secondary antibody only while primary antibody was omitted.

### Statistical analysis

The data are presented as the mean ± SEM. Data analysis was performed by Student's t‐test using StatView 4 (Abacus Concepts, Berkeley, CA, USA). Differences were considered statistically significant when *P *<* *0.05.

Other detailed methods are included in Supporting Information (Data S1).

## Author contributions

JL, YK, TL, and HR were responsible for experimental designs. JL, YK, TL, SJH, HI, and YJH carried out immunohistochemistry, confocal microscopy, quantitative PCR, and Western blot analysis. KEL carried out immunogold staining and electron microscopy. ACM prepared postmortem brain tissues and NWK and HR performed the histological analysis. JL generated and characterized Tet‐inducible cell line. TL prepared primary neuron cultures and measured mitochondrial respiratory function. YK and TL performed FACS analysis. IMJ provided input on the assay of mitochondrial activity. SJU and MH provided p53 constructs and input on the design of the study. HR and NWK wrote the major part of the manuscript. All authors participated in discussing the results and commenting on the manuscript.

## Funding

This study was supported by NIH Grant (R01AG054156‐01 to H.R.). This study was also supported by the National Research Foundation (NRF) Grant (NRF‐2014R1A2A1A11052685 to S.J.U. and NRF‐2016M3C7A1904233), the National Research Council of Science & Technology (NST) Grant (No. CRC‐15‐04‐KIST) from Korea Ministry of Science, ICT and Future Planning (MSIP), and the Flagship Grant (2E26663) from Korea Institute of Science and Technology of South Korea.

## Conflict of interest

None declared.

## Supporting information


**Fig. S1** SIRT3 prevents mito‐p53‐induced cell death.
**Fig. S2** SIRT3 prevents both WT p53‐ and mutant p53 K320Q‐induced cell death.
**Fig. S3** Mitochondrial genome‐encoded transcripts are regulated by mito‐p53.
**Fig. S4** Human mitochondrial p53‐binding elements were determined by p53‐ChIP followed by DNA sequencing.
**Fig. S5** Sirt3 modulates p53‐dependent transcriptional activity of mitochondrial p53‐binding element (Mito‐p53BE)‐driven reporter.
**Fig. S6** SIRT3 is mainly expressed in neuronal cell types.
**Fig. S7** (A) Doxycycline (DOXY) does not affect mitochondrial genome‐encoded gene expression in normal SH‐SY5Y cells.
**Fig. S8**. (A) Western blot shows that SIRT3 reduces caspase‐3 activation induced by p53. Arrow (red) indicates the position of cleaved caspase‐3. (B) SIRT3 significantly reduces the level of cleaved caspase‐3 induced by p53.
**Fig. S9** Knock down of *ND2* and *ND4* decreases SIRT3‐dependent neuroprotective effect against p53.
**Table S1** Information on human normal and AD brain samples.
**Table S2** Primer sequences for qPCR analysis of mitochondrial genes (F, forward; R, reverse).
**Table S3** A list of siRNA sequences for human mitochondria (MT)‐encoded *ND2* and *ND4* genes.
**Data S1** Experimental Procedures.
