# Supplementary material for: SIRT3 deregulation is linked to mitochondrial dysfunction in Alzheimer's disease
Source: Aging Cell. 2017 Nov 11;17(1):e12679. doi: 10.1111/acel.12679 (PMC5771400; doi:10.1111/acel.12679)
Supplement: Supplementary file 1 — Fig. S1 SIRT3 prevents mito‐p53‐induced cell death. Fig. S2 SIRT3 prevents both WT p53‐ and mutant p53 K320Q‐induced cell death. Fig. S3 Mitochondrial genome‐encoded transcripts are regulated by mito‐p53. Fig. S4 Human mitochondrial p53‐binding elements were determined by p53‐ChIP followed by DNA sequencing. Fig. S5 Sirt3 modulates p53‐dependent transcriptional activity of mitochondrial p53‐binding element (Mito‐p53BE)‐driven reporter. Fig. S6 SIRT3 is mainly expressed in neuronal cell types. Fig. S7 (A) Doxycycline (DOXY) does not affect mitochondrial genome‐encoded gene expression in normal SH‐SY5Y cells. Fig. S8. (A) Western blot shows that SIRT3 reduces caspase‐3 activation induced by p53. Arrow (red) indicates the position of cleaved caspase‐3. (B) SIRT3 significantly reduces the level of cleaved caspase‐3 induced by p53. Fig. S9 Knock down of ND2 and ND4 decreases SIRT3‐dependent neuroprotective effect against p53. Table S1 Information on human normal and AD brain samples. Table S2 Primer sequences for qPCR analysis of mitochondrial genes (F, forward; R, reverse). Table S3 A list of siRNA sequences for human mitochondria (MT)‐encoded ND2 and ND4 genes. Data S1 Experimental Procedures. [file ACEL-17-na-s001.doc]

**Supplementary Information to:**

**SIRT3 deregulation is linked to mitochondrial dysfunction in Alzheimer’s disease**

Junghee Lee, Yunha Kim, Tian Liu, Yu Jin Hwang, Seung Jae Hyeon, Hyeonjoo Im,

Victor E. Alvarez, Ann C. McKee, Soojong Um, Manwook Hur,

Inhee Mook-Jung, Neil W. Kowall, and Hoon Ryu

**Supplementary Experimental Procedures**

**Immunohistochemistry**

Immunohistochemistry was performed as previously described (Jo *et al.* 2014). Paraffin-embedded tissues were sectioned in a coronal plane at 10 to 20 μm. The tissue sections were rehydrated, blocked with blocking solution (1% H_2_O_2_), and incubated with anti-SIRT3 antibody (1:500 dilutions) (Upstate Biotech) for 24 hr. After three times of washing, the slides were processed with Vector ABC Kit (Vector Lab). The immunoreactive signals were developed with DAB chromogen (Thermo Fisher Scientific, Meridian, Rockford, USA) and analyzed under a bright field microscopy.

**Heat map analysis of SIRTs in human AD and normal subjects**

To visualize the transcriptional profile of SIRTs, we selected 7 of SIRT genes from Whole RNA-seq data and ran heat map analysis. For mRNA-Seq sample preparation, Illumina standard kits were used according to the manufacturer's protocol. Briefly, 3 μg of each total RNA sample was used for polyA mRNA selection using streptavidin-coated magnetic beads, followed by thermal mRNA fragmentation. The fragmented mRNA was subjected to cDNA synthesis using reverse transcriptase (SuperScript II) and random primers. The cDNA was further converted into double stranded cDNA and, after an end repair process (Klenow fragment, T4 polynucleotide kinase and T4polymerase), was finally ligated to Illumina paired end (PE) adaptors. Size selection was performed using a 2% agarose gel, generating cDNA libraries ranging in size from 200–250 bp. Finally, the libraries were enriched using 10 cycles of PCR and purified by the QIAquick PCR purification kit (Qiagen). The enriched libraries were diluted with Elution Buffer to a final concentration of 10 nM. Each library was run at a concentration of 8 pM on one Genome Analyzer (GAIIx) lane using 53 bp sequencing. Reads were then processed and aligned to the mouse genome UCSC build mm9 using GSNAP (Wu & Nacu 2010). GSNAP uses the normalized RNA-Seq 8 fragment counts to measure the relative abundances of transcripts. The unit of measurement is Reads Per Kilobase of exon per Million fragments mapped (RPKM) (Mortazavi *et al.* 2008).

**Immunofluorescence staining and confocal microscopy**

DOXY-inducible Mito-p53 cells or primary cortical neurons cultured on glass coverslips coated with 100μg/ml Poly-D-lysine in 24-well plate. After cells were fixed with 4% paraformaldehyde, and permeabilized with 0.2% Triton X-100, cells were stained with specific primary antibodies and with fluorescence-tagged secondary antibodies. Coverslips were mounted using Gel/Mount (Biomeda, CA). Cells were observed by confocal microscopy (Olympus FV10i, Tokyo, Japan).

**Western blot analysis**

Thirty g of protein was subjected to SDS-PAGE (10%) and blotted with anti-SIRT3 (Upstate Biotech.), anti-Sp1 [sc-59 (PEP2)], Sp3 [sc-664 (D-20)], and Sp4 [sc-645 (V-20)] (Santa Cruz Biotech.) antibody. TOM20 mouse monoclonal antibody [sc-17764, F-10] ([Santa Cruz Biotechnology](https://www.scbt.kr/) Inc.) was used at 1:100 dilution. Protein loading was controlled by probing for alpha-tubulin (Sigma) or beta-actin (Sigma) on the same membrane.

**Constructs of Sirt3 (full length and truncated) and GST fusion protein purification**

Generation of GST-Sirt3 full length and truncated constructs has been described previously (Onyango *et al.* 2002). Expression of the fusion proteins was confirmed by growing 5ml cultures followed by induction with IPTG (1mM final concentration) at OD_600_ ~0.6. Clones showing maximal expression of our fusion proteins were selected for doing larger cultures for protein purification. Typically, 300 ml cultures were set up for each of the sirt3 constructs and grown (at 37˚C, 250 rpm) till an OD_600_ of 0.6 was reached at which induction with IPTG was done and the cells were harvested by centrifugation (5000 rpm for 15 min at 4˚C) after 4h of induction. Cells were resuspended in 10ml lysis buffer (chilled on ice) containing 50mM Tris-HCl, pH7.4, 350mM NaCl, 5mM EDTA, 1% Triton X-100 and 0.5% NP-40. The lysis buffer was supplemented with protease inhibitors (Leupeptin, Pepstatin, NaF, PMSF, Na_3_VO_4_, MG132, DTT, NEM) to minimize protein degradation. The lysis was performed using a combination of freeze-thaw (in dry-ice & methanol slurry) and sonication. The lysate was then centrifuged (15,000 rpm/15min at 4˚C) and the soluble fraction (supernatant) was collected. The supernatant was incubated with 700 or 500μl of Glutathione-Sepharose 4B (Amersham Pharmacia Biotech, Sweden) with gentle rocking for 3h at 4˚C. After incubation, the fusion proteins bound to the beads were separated from unbound fraction by centrifugation at 3000 rpm for 10min. The unbound supernatant was removed and the beads were washed with lysis buffer twice followed by two washes with PBS. For analysis of the purity, 25μl of the beads were boiled in SDS-PAGE sample buffer followed by SDS-PAGE and staining with Coomassie Blue.

**Cell apoptosis assay and mitochondrial dysfunction assay**

DOXY-inducible Mito-p53 cells were cultured in DMEM medium with 10% FBS and 1% penicillin/streptomycin in 24-well plate. After 24h incubation, transfection was performed using lipofectamine 2000 according to manufacturer’s instruction, and/or cells were directly treated with DOXY for the expected time. For Annexin V / PI cell death assays, the Annexin-V-FITC and PI Apoptosis Detection Kit (BD Biosciences) was used followed by FACS analysis (FACSCalibur, BD, CA, USA) according to the manufacturer’s instructions. For DCF-DA (Invitrogen), JC-1 staining and MitoTracker staining, after harvesting and washing cell with PBS, cells were stained with JC-1 (Invitrogen) or MitoTracker (Invitrogen) Red for 15min. Cells were washed one time with binding buffer and measured by flow cytometry and/or captured by fluorescence microscopy.

**Measurement of oxygen consumption rate**

Mito-p53 cells were cultured in specific 24-well microplate from Seahorse (MA, USA). After cells were ready for analysis, medium was replaced with a respiration buffer (Seahorse, MA, USA) containing 225 mM mannitol, 75 mM sucrose, 10 mM KCl, 10 mM Tris-HCl and 5 mM KH_2_PO_4_ at pH 7.2. Cells respiration was then measured using a Seahorse XF24 analyzer (MA, USA). The XF Cell Mito Stress Test Kit (MA, USA) was also used to measure key parameters of mitochondrial function in microplates including basal respiration, ATP turnover and proton leak in the presence of the mitochondrial inhibitor oligomycin (0.5 µM), and maximal respiration in the presence of the mitochondrial uncouple FCCP (1 µM), and spare respiratory capacity in the presence of antimycin A (1 µM) and rotenone (1 µM).

**Chromatin Immunoprecipitation (ChIP) and quantitative PCR assay**

Brain tissue fractions were crossed-linked with 1% formaldehyde for 20 min at room temperature. The lysates were sonicated six times for 20 sec using a Branson Sonifier 450 (Branson Ultrasonics Corp., Danbury, CT). After centrifugation, the supernatant was diluted in ChIP dilution buffer and incubated overnight at 4 °C with anti-p53 antibody. Immune complexes were recovered by the addition of 60 ml of salmon sperm DNA/protein A agarose-50% slurry and incubation for 2 h at 4 °C with rotation. The beads were pelleted and washed with low and high salt buffer, LiCl buffer and finally twice with TE buffer. Immune complexes were then eluted by incubation for 15 min at 37 °C with fresh elution buffer (1% SDS, 0.1 M NaHCO_3_). To reverse the cross-linking of DNA, 10 ml of 5M NaCl were added to the combined elute (150 ml) and incubated overnight at 65 °C. DNA was purified using a QIA quick DNA purification spin column and eluted in 50 ml of nuclease free water. Quantitative PCR amplification was carried out for 40 cycles. The ChIP-qPCR primers were as following: Human 12S rRNA; forward, 5’-CTACTCGCCTCTACGTCTACC-3’ and reverse, 5’-CCCAGAACGCCATGTTCC-3’ Human ND5; 5’-GCCTTCTTCAAAGCCATACT-3’ and reverse, 5’- ATTCCTGCTAATGCTAGGCT -3’.

**Mitochondrial p53 binding element (BE)-driven reporter assay**

SH-SY5Y cells were cultured in 48-well plates at a density of 5×10^4^ cells per well, and the next day, cells were transfected with pGL4 or pGL4-mito-p53 BE1 in various constructs under specific condition. The luciferase activity was measured with dual luciferase assay kit 24 h after the transfection. The reporter activities were normalized to *Renilla* luciferase activity or to protein concentrations.

**Cell viability and mitochondrial activity assay**

Cell viability was quantified using MTT (Sigma, M2128) as previously described (Berridge and Tan 1993; Kim *et al.* 2013).

**References**

Berridge MV, Tan AS (1993). Characterization of the cellular reduction of 3-(4,5-dimethylthiazol-2-yl)-2,5-diphenyltetrazolium bromide (MTT): subcellular localization, substrate dependence, and involvement of mitochondrial electron transport in MTT reduction. *Arch Biochem Biophys* **303**,474–482.

Jo S, Yarishkin O, Hwang YJ, Chun YE, Park M, Woo DH, Bae JY, Kim T, Lee J, Chun H, Park HJ, Lee da Y, Hong J, Kim HY, Oh SJ, Park SJ, Lee H, Yoon BE, Kim Y, Jeong Y, Shim I, Bae YC, Cho J, Kowall NW, Ryu H, Hwang E, Kim D, Lee CJ (2014). GABA from reactive astrocytes impairs memory in mouse models of Alzheimer's disease. *Nat Med*. **20**, 886-896.

Mortazavi A, Williams BA, McCue K, Schaeffer L, Wold B (2008). Mapping and quantifying mammalian transcriptomes by RNA-Seq. *Nat Methods*. **5**, 621-628.

Onyango P, Celic I, McCaffery JM, Boeke JD, Feinberg AP (2002). SIRT3, a human SIR2 homologue, is an NAD-dependent deacetylase localized to mitochondria. *Proc Natl Acad Sci U S A*. **99**, 13653-13658.

Wu TD, Nacu S (2010). Fast and SNP-tolerant detection of complex variants and splicing in short reads. *Bioinformatics*. **26**, 873-881.

**Supplementary Table 1. Information on human normal and AD brain samples.**

| **Case** | **Age** | **Sex** | **Braak stage** |
| --- | --- | --- | --- |
| Normal 1 | 87 | Female | I |
| Normal 2 | 88 | Male | I |
| Normal 3 | 86 | Male | II |
| Normal 4 | 87 | Female | II |
| Normal 5 | 67 | Male | I |
| Normal 6 | 82 | Male | I |
| Normal 7 | 61 | Male | I |
| Normal 8 | 101 | Female | I |
| Normal 9 | 89 | Male | III |
| Normal 10 | 68 | Male | I |
| Normal 11 | 78 | Female | I |
| Normal 12 | 89 | Male | III |
| Normal 13 | 70 | Male | I |
| AD 1 | 82 | Male | V |
| AD 2 | 79 | Female | VI |
| AD 3 | 70 | Male | VI |
| AD 4 | 59 | Male | VI |
| AD 5 | 80 | Female | V |
| AD 6 | 92 | Male | V |
| AD 7 | 90 | Female | V |
| AD 8 | 100 | Male | V |
| AD 9 | 75 | Male | V |
| AD 10 | 83 | Male | VI |
| AD 11 | 79 | Female | VI |
| AD 12 | 89 | Male | IV |
| AD 13 | 69 | Male | VI |

**Supplementary Table 2. Primer sequences for qPCR analysis of mitochondrial genes (F, forward; R, reverse).**

| **Gene** | **Sequence** |
| --- | --- |
| Human *12srRNA* | F- GGA ACA AGC ATC AAG CA |
|  | R- CGG TAT ATA GGC TGA GCA |
| Human *ATPase6* | F- CAA CCG ACT AAT CAC CA |
|  | R- GCT TGG ATT AAG GCG ACA |
| Human *ATPase8* | F- GCC CCA ACT AAA TAC TAC CGT A |
|  | R- GGG GCA ATG AAT GAA GCG AA |
| Human *COX1* | F- CTC TAA GCC TCC TTA TTC GA |
|  | R- CAG CTA GGA CTG GGA GA |
| Human *COX2* | F- AAG ACG CTA CTT CCC CTA |
|  | R- GTT AGA CGT CCG GGA A |
| Human *COX3* | F- CAT ACT AGG CCT ACT AAC AA |
|  | R- GTG AGG AAA GTT GAG CCA A |
| Human *CYTB* | F- TAT CTG CCT CTT CCT ACA CA |
|  | R- GTG TTT AAG GGG TTG GCT A |
| Human *ND3* | F- ACC ACA ACT CAA CGG CTA |
|  | R- TAG TCA CTC ATA GGC CAG A |
| Human *ND4L* | F- CTA GTA TAT CGC TCA CAC CTC A |
|  | R- TAC GTA GTC TAG GCC ATA TGT G |
| Human *ND5* | F- AGC CTA GCA TTA GCA GGA |
|  | R- GCA GGT TTT GGC TCG TA |
| Human *ND6* | F- AGC GAT GGC TAT TGA GGA |
|  | R- CAA TAG GAT CCT CCC GAA |

**Supplementary Table 3. A list of siRNA sequences for human mitochondria (MT)-encoded *ND2* and *ND4* genes.**

| **Name** | **siRNA Sequence** |
| --- | --- |
| siRNA Negative Control | Sense : UUCUCCGAACGUGUCACGUTT  Anti-sense : ACGUGACACGUUCGGAGAATT |
| siRNA MT-*ND2* (Human)-A | Sense : GCAAGCAACCGCAUCCAUATT  Anti-sense : UAUGGAUGCGGUUGCUUGCTT |
| siRNA MT-*ND2* (Human)-B | Sense : CCGUCAUCUACUCUACCAUTT  Anti-sense : AUGGUAGAGUAGAUGACGGTT |
| siRNA MT-ND2 (Human)-C | Sense : CCUGAGUAGGCCUAGAAAUTT  Anti-sense : AUUUCUAGGCCUACUCAGGTT |
| siRNA MT-*ND4* (Human)-A | Sense : GCAAGCCAACGCCACUUAUTT  Anti-sense : AUAAGUGGCGUUGGCUUGCTT |
| siRNA MT-*ND4* (Human)-B | Sense : CCCUAUACUCCCUCUACAUTT  Anti-sense : AUGUAGAGGGAGUAUAGGGTT |
| siRNA MT-*ND4* (Human)-C: | Sense : CCUCGUAGUAACAGCCAUUTT  Anti-sense : AAUGGCUGUUACUACGAGGTT |

**Supplemental Figure Legends**

**Fig. S1** SIRT3 prevents mito-p53-induced cell death. (A) Phase contrast microscopic images show that induction of mito-p53 leads to cell death. (B) Microscopic images show that SIRT3 expression rescued mito-p53-induced cell death. Doxycycline (DOXY) was treated to induce mito-p53 for 42 h and cell morphology was examined under a phase contrast microscopy. (C) Annexin V/PI staining analyzed by flow cytometry shows that mutant SIRT3 N87A did not prevent mito-p53 induced cell death. The bar graph represents the mean ± SEM of three independent experiments (n = 3; ****P* < 0.001). (D) JC-1 staining analyzed by flow cytometry shows that mutant SIRT3 N87A did not prevent mito-p53-induced mitochondrial depolarization due to the opening of mitochondrial permeability transition pore. R1 area represents a proportion of the JC-1 monomer that is produced by alteration of mitochondrial potential. The bar graph represents the mean ± SEM of three independent experiments (n = 3; **P* < 0.05, ***P* < 0.001).

**Fig. S2** SIRT3 prevents both WT p53- and mutant p53 K320Q-induced cell death. (A) Annexin/ PI staining followed by flow cytometry shows that SIRT3 ameliorated p53-induced cell death. Cells (SH-SY5Y) were transiently transfected with WT p53 and mutant p53 K320Q (acetylation site mimetic mutant) in the presence or absence of SIRT3 for 36hr. A representative scatter image is shown among five independent experiments. (B) SIRT3 significantly ameliorated p53-induced cell death in terms of Annexin V activity. This data is originated from (A). The bar graph represents the mean ± SEM of five independent experiments (n=5; significantly different from control at **P* < 0.05 and ***P* < 0.01, significantly different from p53-transfected group at ^##^*P* < 0.01).

**Fig. S3** Mitochondrial genome-encoded transcripts are regulated by mito-p53. Quantitative PCR was performed to determine mRNA levels of human *ND5*, *16S rRNA*, *ND6*, *COX1*, *COX3*, *Cytochrome b*, *ND3*, *ATPase6*, *COX2*, *ATPase8*, and *ND4L* genes in response to mito-p53 activation. The RNA was isolated from mito-p53 inducible SH-SY5Y cells treated with DOXY for 0, 6, and 12h. The bar graph represents the mean ± SEM of four independent experiments (n = 4; significantly different from 0 hr at **P* < 0.05).

**Fig. S4** Human mitochondrial p53-binding elements were determined by p53-ChIP followed by DNA sequencing. (A) Sequencing chromatograms show that p53-ChIP DNA samples from human AD brains contain mitochondrial *ND2* and *12S rRNA* genes. (B) DNA sequences from p53-ChIP were aligned with human mitochondrial *ND2* and *12S rRNA* genes. The alignment was generated using Multalin software (<http://multalin.toulouse.inra.fr/multalin/multalin.html>). (C) Mitochondrial *ND2* and *12S rRNA* DNA sequences possesses potential consensus binding sites for p53. (D) Mito-p53 binding to DNA of mitochondrial *12S rRNA* gene was significantly increased by DOXY treatment for 12h while its occupancy to *ND5* gene was not significantly affected in Tet-inducible Mito-p53 SH-SY5Y cells. The DNA occupancy of mito-p53 at *12S rRNA* and *ND5* gene was quantified by using qPCR. *C*t values of immunoprecipitated DNA were normalized to *C*t values obtained from input DNA (n = 3; **P* < 0.05).

**Fig. S5** Sirt3 modulates p53-dependent transcriptional activity of mitochondrial p53-binding element (Mito-p53BE)-driven reporter. (A) A scheme shows human mitochondria DNA sequences harboring potential consensus p53 binding elements. p53 can bind to the double-stranded mitochondrial DNA with sites comprising several copies of half-site containing 10 base pairs with a sequence of PuPu-PuC(A/T)(T/A)GPyPyPy (Pu, purines and Py, pyrimidines). (B) A scheme shows a construction of mitochondrial consensus p53BE-driven reporter vector using pGL3E-firefly luciferase vector. (C) p53 overexpression significantly increased Mito-p53-BE1 reporter (1x and 2x BEs) activity. Cells (SH-SY5Y) were transiently co-transfected with pGL3E empty vector or pGL3E-Mito-p53BE1 with or without p53 (100 ng/ml). Luciferase activity was normalized to the protein concentration of each sample. Data are the mean ± SEM of three separate experiments. (n=3; **P* < 0.05, ***P* < 0.001). (D) p53 induced Mito-p53BE1 reporter activity in a dose-dependent manner. Cells were transiently cotransfected with pGL3E or pGL3E-Mito-p53BE1 in the presence of p53. Data are the mean ± SEM of three separate experiments (n=3; ***P<*0.001). (E) SIRT3 reduced p53-induced Mito-p53BE1 reporter activity. Data are the mean ± SEM of three separate experiments (n=3; ***P*<0.001).

**Fig. S6** SIRT3 is mainly expressed in neuronal cell types. A coronal section of mouse brain was stained with NeuN (green), a neuronal marker, and SIRT3 (red) followed by DAPI counter staining. The SIRT3 immunoreactivity was found as cytosolic puncta structures (mitochondrial localization) in NeuN-positive neuronal cells. Scale bar (white): 10μm.

**Fig. S7** (A) Doxycycline (DOXY) does not affect mitochondrial genome-encoded gene expression in normal SH-SY5Y cells. DOXY has no significant effects on the expression of *ND2* and *ND4* genes. Gene expression levels were normalized to *12S RNA*. Melting curve (left) and amplification (ΔCT) curve (right) show that qPCR primers for *ND2, ND4* and *12S RNA* gene are working properly. The bar graph represents the mean ± SEM of three separate experiments (n=3). (B) MTT assay shows that DOXY treatment affects mitochondrial electron transport in SH-SY5Y cells*.* Low doses of DOXY decrease mitochondrial activity. The inhibitory effect of DOXY on the mitochondrial activity is sustained at high doses and is similar to low doses. Significantly different at **P*<0.05.

**Fig. S8**. (A) Western blot shows that SIRT3 reduces caspase-3 activation induced by p53. Arrow (red) indicates the position of cleaved caspase-3. (B) SIRT3 significantly reduces the level of cleaved caspase-3 induced by p53. The bar graph represents the mean ± SEM of three separate experiments (n=3). Significantly different from control at **P*<0.05 and from Mito-p53 at ^#^*P*<0.01.

**Fig. S9** Knock down of *ND2* and *ND4* decreases SIRT3-dependent neuroprotective effect against p53. (A) Melting curve (top) and amplification (ΔCT) curve (middle) show that qPCR primers of *ND2* gene is working properly. *siRNA ND2* significantly reduces the mRNA level of *ND2* (bottom left). The bar graph represents the mean ± SEM of three separate experiments (n=3). Significantly different at ***P*<0.01. (B) Melting curve (top) and amplification (ΔCT) curve (middle) show that qPCR primers of *ND4* gene is working properly. *siRNA ND4* significantly reduces the mRNA level of *ND2* (bottom left). The bar graph represents the mean ± SEM of three separate experiments (n=3). Significantly different at ***P*<0.01. (C) Knock down of *ND2* and *ND4* by siRNAs decreased SIRT3-dependent cell viability in SH-SY5Y cells. The cell viability was measured by MTT assay. The data represents the mean ± SEM of three separate experiments (n=3). Significantly different at **P*<0.05, ***P*<0.01, ^#^*P*<0.05, and ^##^*P*<0.01. (D) Western blot shows that knock down of *ND2* and *ND4* by siRNAs increased the level of cleaved caspase-3 in SH-SY5Y cells. Arrow (red) indicates the position of cleaved caspase-3.

**Supplementary Figures**

**Fig. S1**

**
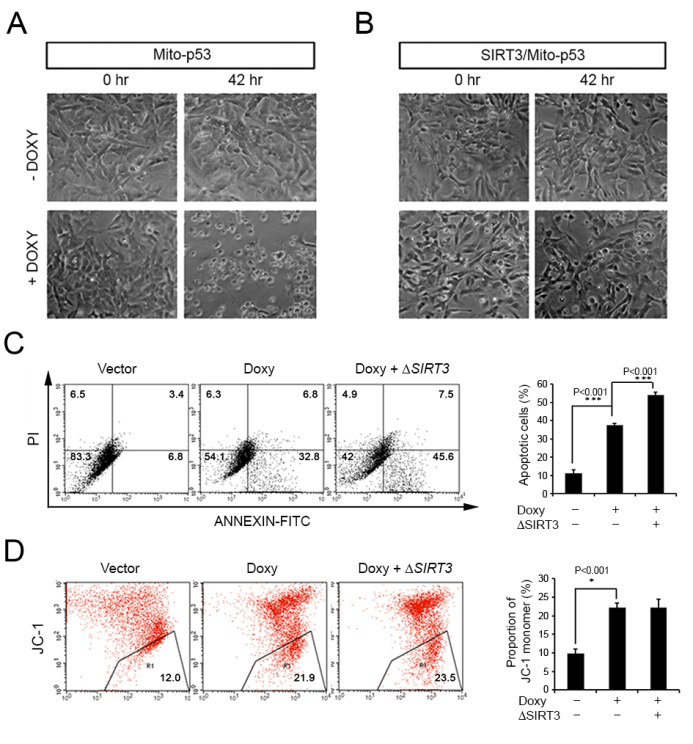
**

**Fig. S2**

**
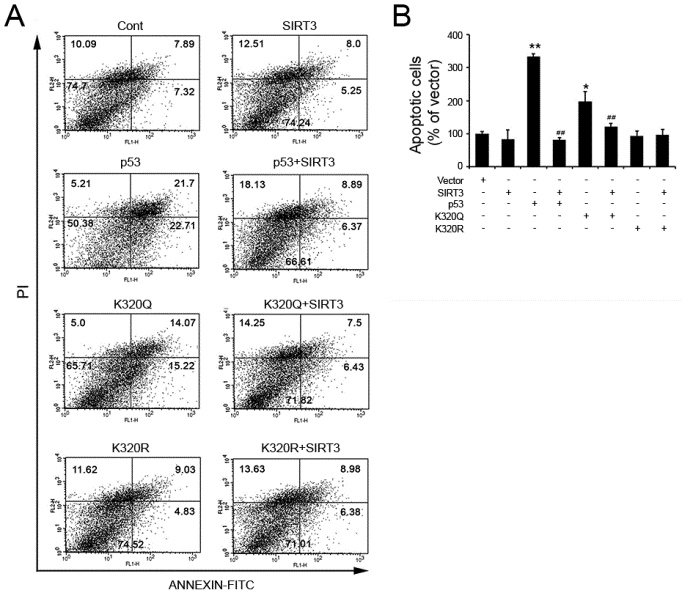
**

**Fig. S3**

**
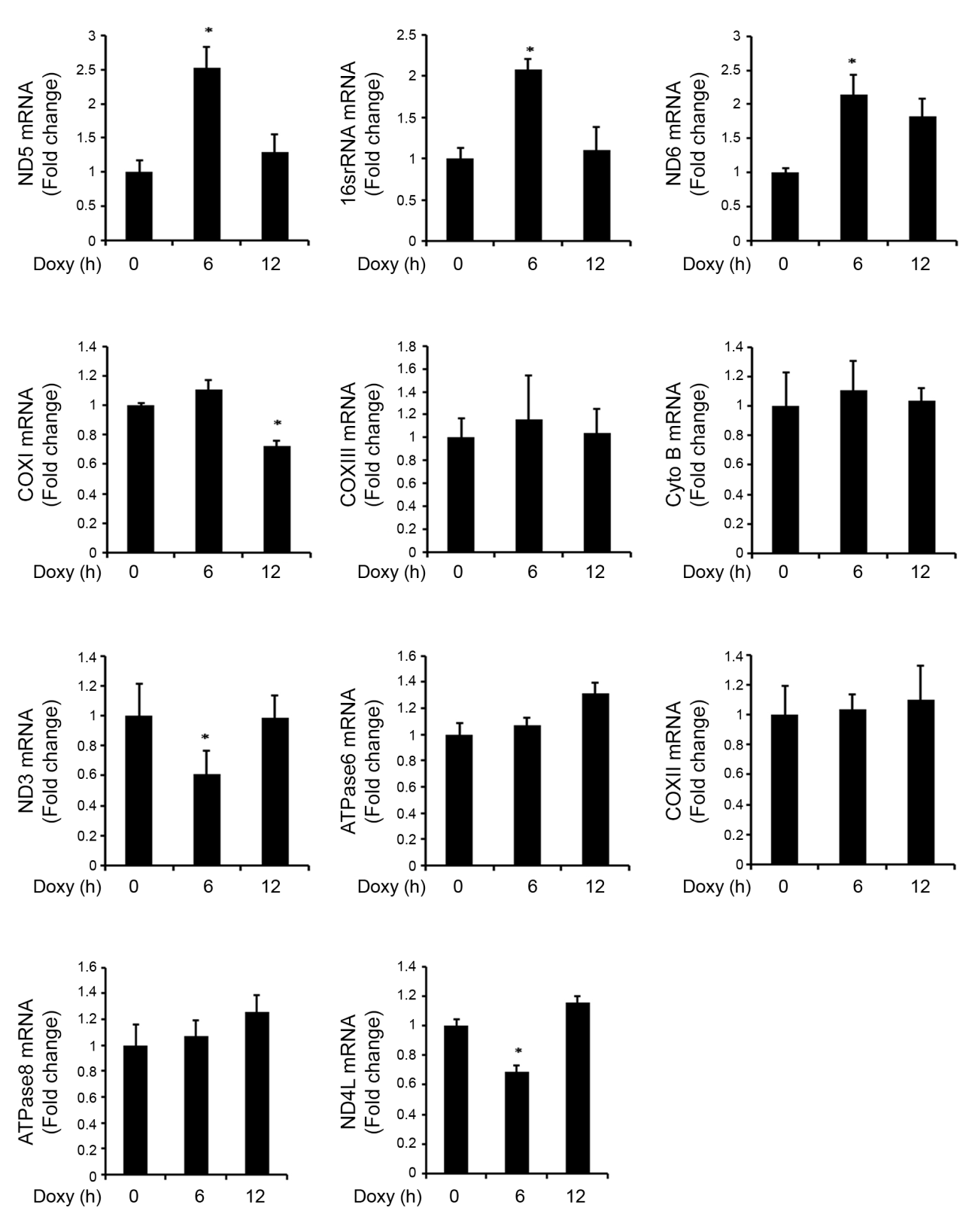
**

**Fig. S4**

**
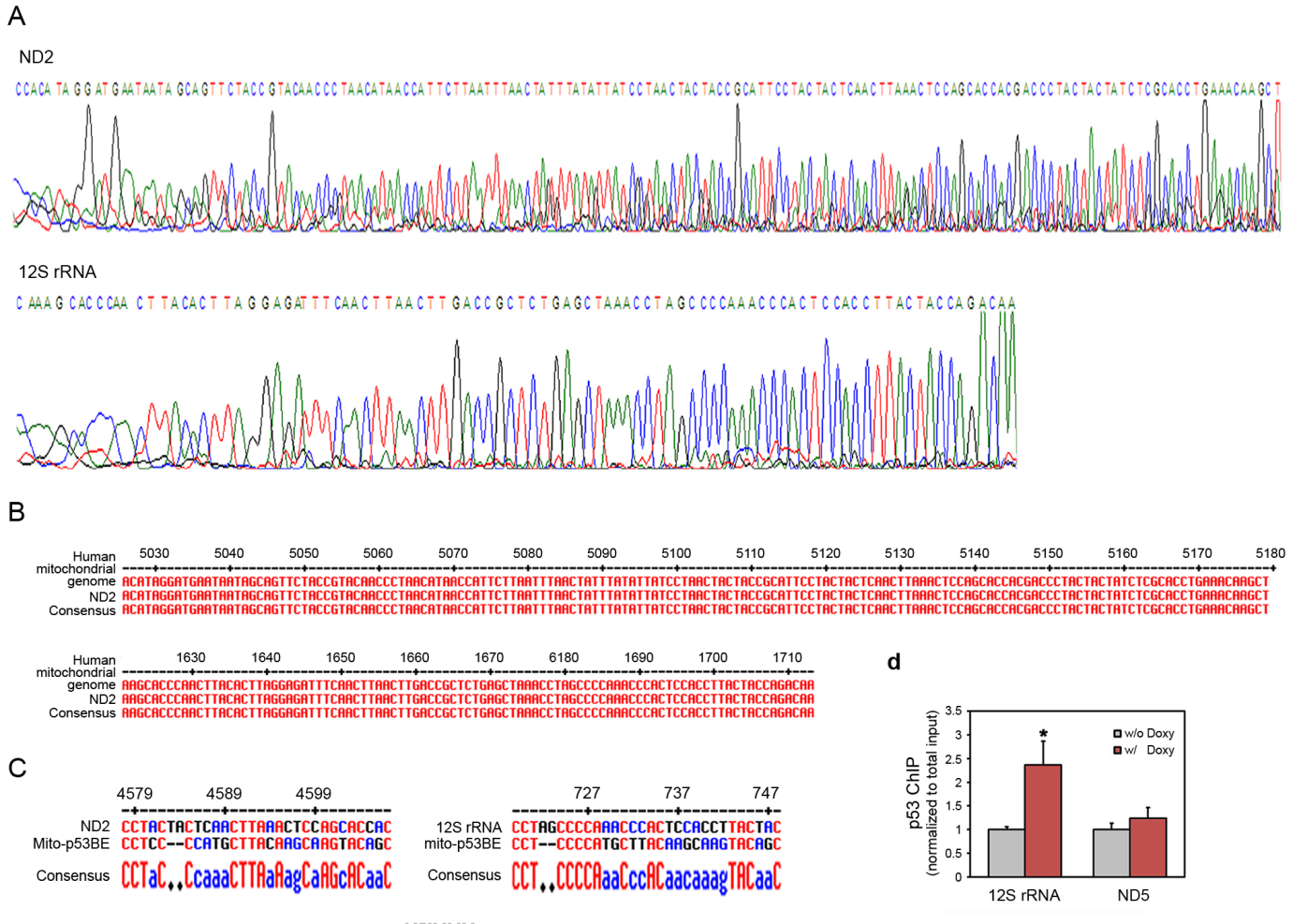
**

**Fig. S5**


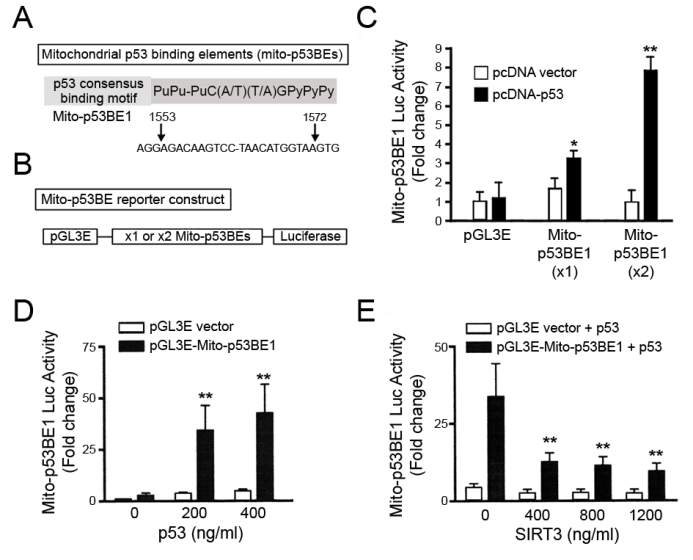


**Fig. S6**


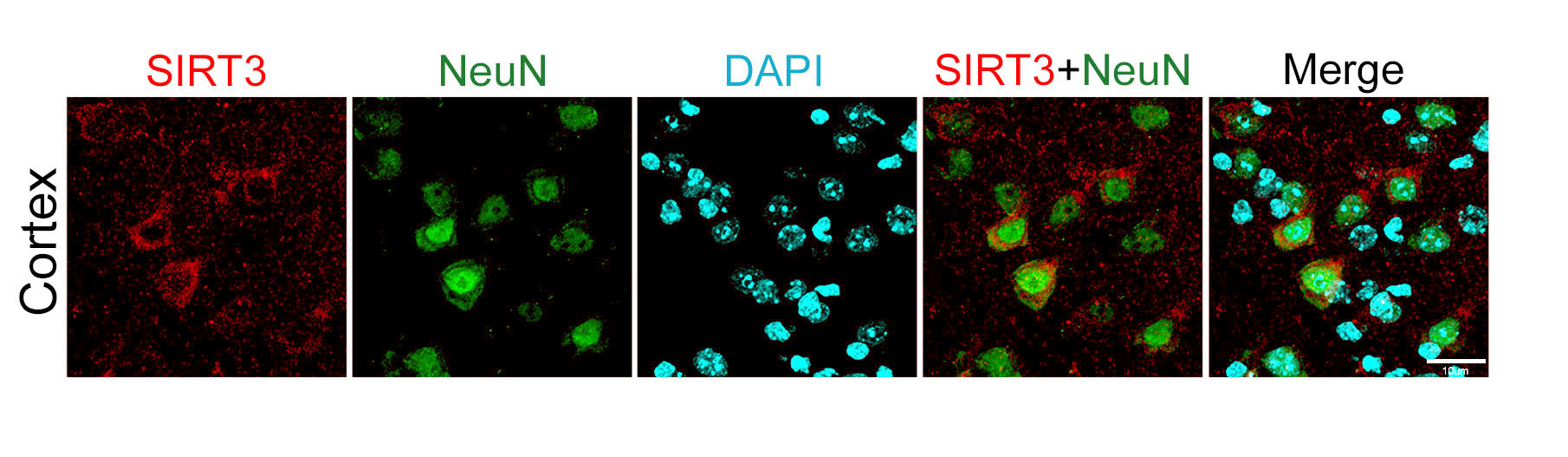


**Fig. S7**

**
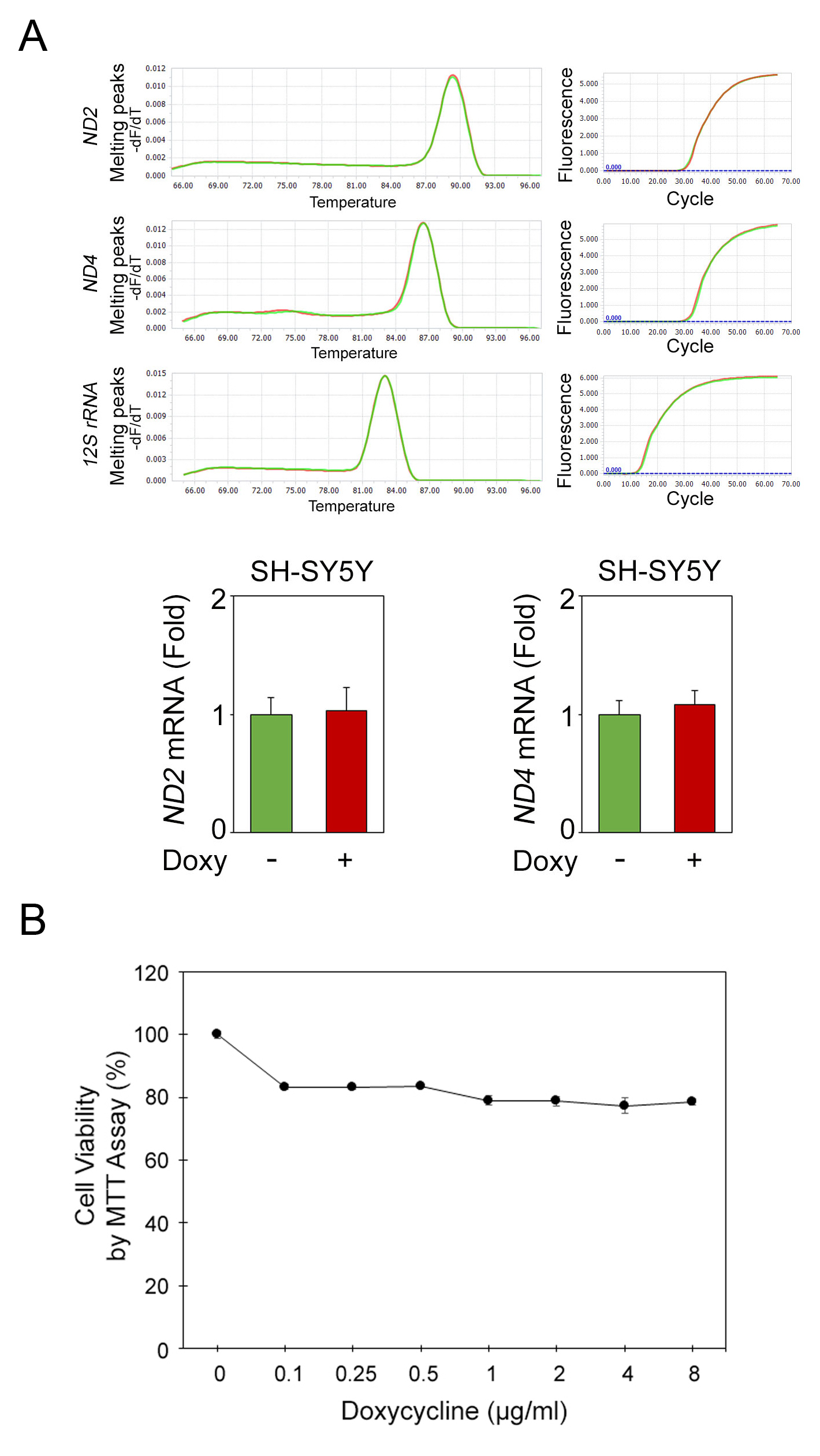
**

**Fig. S8**

**
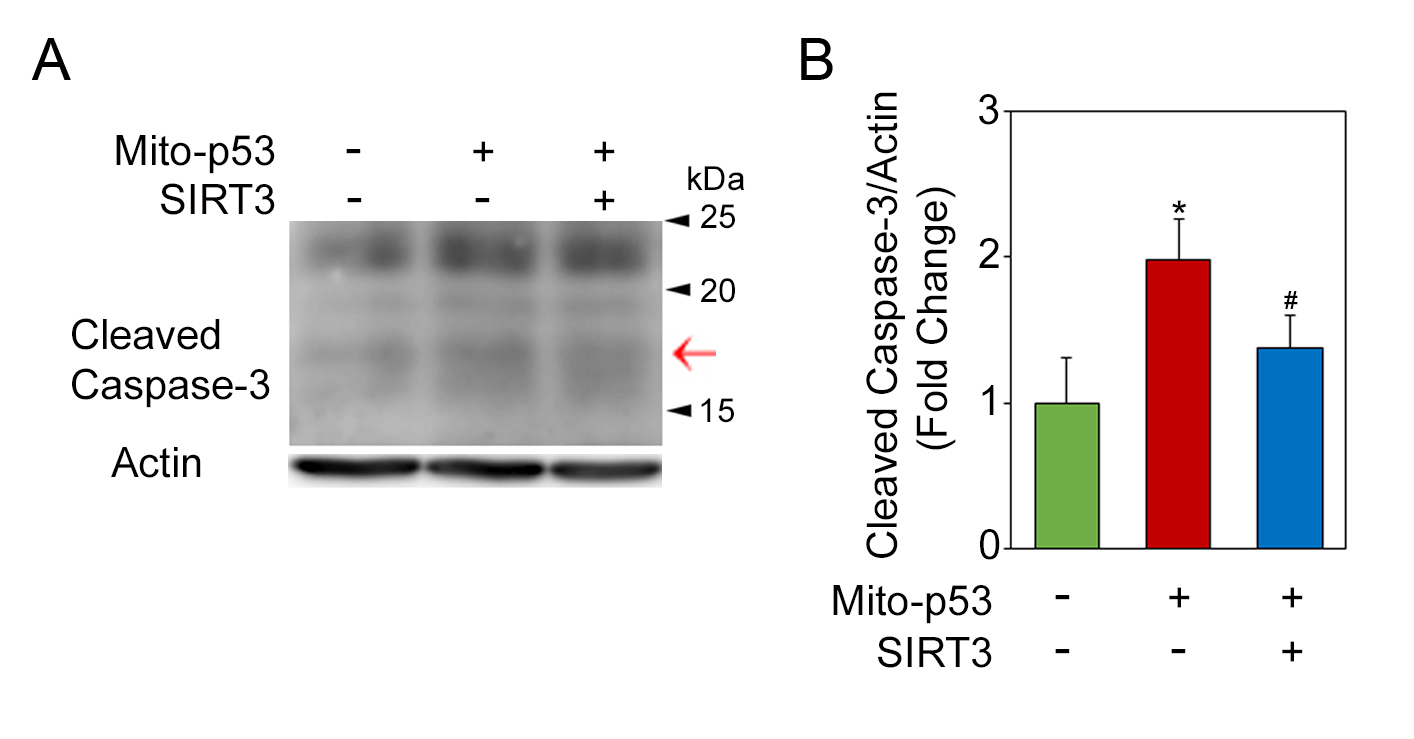
**

**Fig. S9**

**
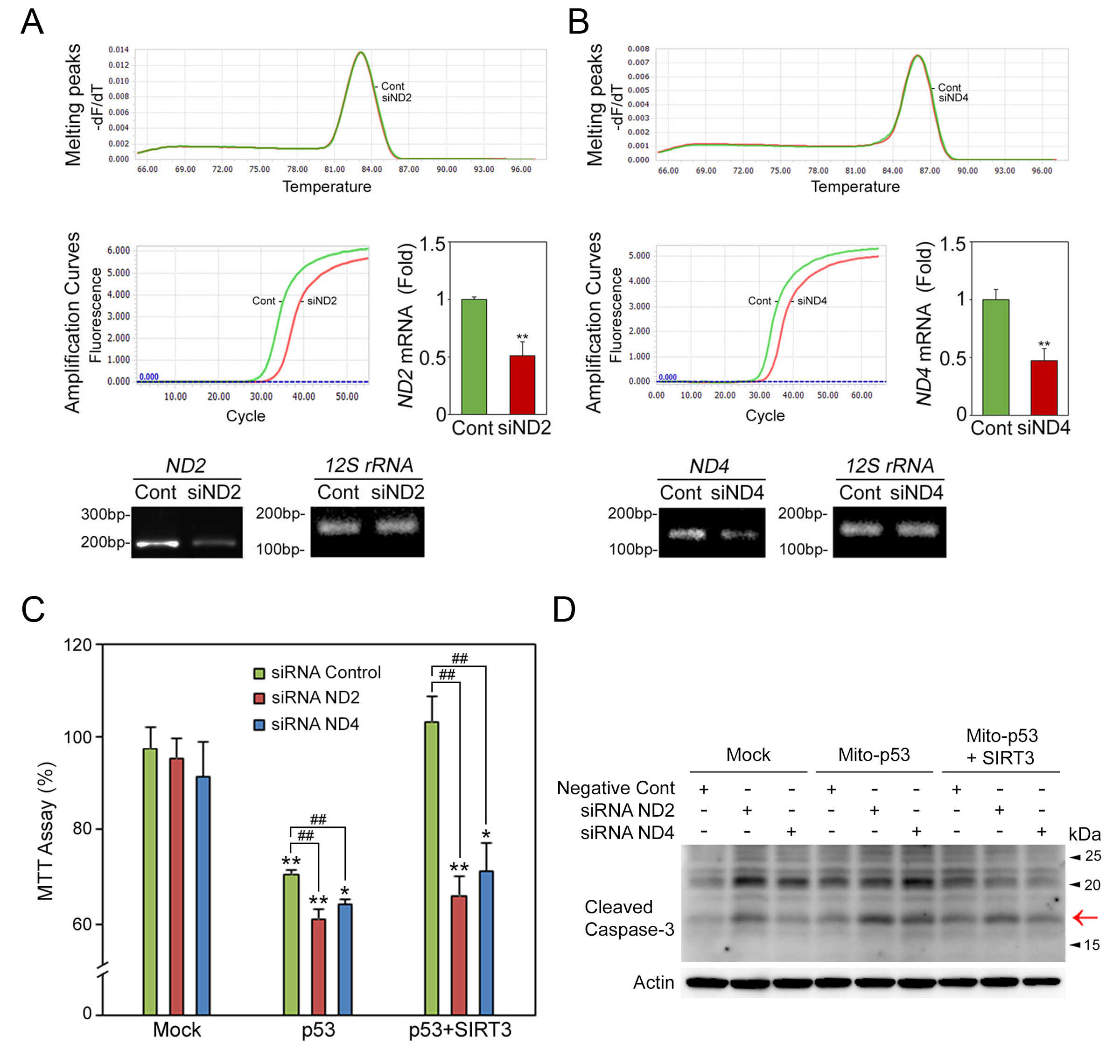
**
